# Rapid and efficient room-temperature serial synchrotron crystallography using the CFEL TapeDrive

**DOI:** 10.1107/S2052252522010193

**Published:** 2022-10-31

**Authors:** Kara A Zielinski, Andreas Prester, Hina Andaleeb, Soi Bui, Oleksandr Yefanov, Lucrezia Catapano, Alessandra Henkel, Max O. Wiedorn, Olga Lorbeer, Eva Crosas, Jan Meyer, Valerio Mariani, Martin Domaracky, Thomas A. White, Holger Fleckenstein, Iosifina Sarrou, Nadine Werner, Christian Betzel, Holger Rohde, Martin Aepfelbacher, Henry N. Chapman, Markus Perbandt, Roberto A. Steiner, Dominik Oberthuer

**Affiliations:** a Center for Free-Electron Laser Science CFEL, Deutsches Elektronen-Synchrotron DESY, Notkestr. 85, 22607 Hamburg, Germany; b Institute for Medical Microbiology, Virology and Hygiene, University Medical Center Hamburg-Eppendorf, Martinistrasse 52, 20246 Hamburg, Germany; c Institute of Biochemistry and Molecular Biology, Laboratory for Structural Biology of Infection and Inflammation, University of Hamburg, c/o DESY, Building 22a, Notkestr. 85, 22603 Hamburg, Germany; d Randall Centre of Cell and Molecular Biophysics, King’s College London, United Kingdom; e MRC Laboratory of Molecular Biology, Francis Crick Avenue, Cambridge CB2 0QH, United Kingdom; f Deutsches Elektronen-Synchrotron DESY, Notkestr. 85, 22607 Hamburg, Germany; g Hamburg Centre for Ultrafast Imaging, Universität Hamburg, Luruper Chaussee 149, 22761 Hamburg, Germany; h Department of Physics, University of Hamburg, Luruper Chaussee 149, 22761 Hamburg, Germany; i Department of Biomedical Sciences, University of Padova, via Ugo Bassi 58/B, Padova 35131, Italy; Uppsala University, Sweden

**Keywords:** serial crystallography, partial reflections, protein structures, sample delivery, room temperature, CFEL TapeDrive, *K. pneumoniae* CTX-M-14, *N. haematococca* GH11 xylanase, *A. flavus* urate oxidase

## Abstract

Serial crystallography at conventional synchrotron light sources (SSX) offers the possibility to routinely collect data at room temperature using micrometre-sized crystals of biological macromolecules. However, SSX data collection is not yet as routine and currently takes significantly longer than the standard rotation series cryo-crystallography. Thus, its use for high-throughput approaches, such as fragment-based drug screening, where the possibility to measure at physio­logical temperatures would be a great benefit, is impaired. On the way to high-throughput SSX using a conveyor belt based sample delivery system – the CFEL TapeDrive – with three different proteins of biological relevance (*Klebsiella pneumoniae* CTX-M-14 β-lactamase, *Nectria haematococca* xylanase GH11 and *Aspergillus flavus* urate oxidase), it is shown here that complete datasets can be collected in less than a minute and only minimal amounts of sample are required.

## Abstract 2

Using the CFEL TapeDrive, minimization of sample consumption and data collection times needed for serial synchrotron crystallography (SSX) were achieved and the first room-temperature structures of two enzymes are described as well as an assessment of the benefit of SSX for a radiation-sensitive protein.

## 1. Introduction

Unlike fixed-axis macromolecular X-ray crystallography (MX), where typically a single crystal is rotated to collect a complete set of diffraction data, serial crystallography uses thousands of microcrystals presented to the beam at random orientations to achieve a full dataset (Chapman *et al.*, 2011 ▸; Barends *et al.*, 2022 ▸; Orville, 2020 ▸). Now, more than ten years after the first successful serial crystallography experiments at the AMO experimental station at LCLS (SLAC, USA) (Chapman *et al.*, 2011 ▸), this approach is used not only at X-ray free-electron laser sources (XFELs), but also increasingly frequently at third- and fourth-generation synchrotron radiation sources (Stellato *et al.*, 2014 ▸; Beyerlein *et al.*, 2017 ▸; Schulz *et al.*, 2018 ▸; Botha *et al.*, 2018 ▸, 2015 ▸; Weinert *et al.*, 2019 ▸, 2017 ▸; Martin-Garcia *et al.*, 2017 ▸).

Although serial synchrotron X-ray crystallography (SSX) does not benefit from the ‘diffract-before-destroy’ principle (Chapman *et al.*, 2014 ▸) exploited by XFEL serial femtosecond crystallography (SFX), the wide availability of macromolecular crystallography experimental stations at synchrotrons worldwide has spurred interest to develop alternative approaches and explore possibilities for serial measurements. The X-ray intensity at third- and fourth-generation synchrotron sources such as PETRA III, NSLS II, MAX IV, APS, ESRF, SLS, SPring-8 and Diamond Light Source allows for exposure times of micrometre-sized crystals in the low millisecond to microsecond range (Nanao *et al.*, 2022 ▸). These are much longer than those at XFELs that are typically on the order of tens of femtoseconds. While this limits the time-resolution of experimental triggers for SSX compared with SFX, the use of polychromatic X-rays (‘pink beam’) with 100 ps-long pulses has also been successful for the serial Laue approach (Meents *et al.*, 2017 ▸). Shorter exposure times offer the benefit that sample delivery methods used at XFELs, including liquid jets, can in principle be used for serial Laue crystallography, thus enabling fast mix-and-diffuse experiments for time-resolved enzymology studies. However, serial Laue crystallography experiments are rather time-consuming partly because automated data-processing tools are still lacking, especially for indexing and integration steps. Additionally, there are only a handful of experimental stations currently in operation worldwide [BioCARS/Advanced Photon Source (APS), ID9 and ID29/European Synchrotron Radiation Facility (ESRF)] that have the capability to perform such experiments. On the other hand, there is a wealth of monochromatic MX beamlines in operation and although time resolution is limited to the sub-millisecond range, due to the at least 1000-fold reduction in X-ray intensity compared wiith polychromatic X-rays, this still allows for many enzymatic reactions to be followed by time-resolved crystallography, either by light activation of photocaged substrates (Mehrabi, Schulz, Dsouza *et al.*, 2019 ▸), mix-and-diffuse methods (Beyerlein *et al.*, 2017 ▸; Mehrabi, Schulz, Agthe *et al.*, 2019 ▸) or other triggering methods.

For SSX, crystals can be as small as the X-ray spot size, which with the advent of diffraction-limited sources and their loss-free achievable beam sizes, will allow for diffraction data collection from sub-micrometre-sized crystals, enabling more advanced mix-and-diffuse methods, not only for time-resolved measurements, but also for high-output drug-design studies. However, compared with SFX, the longer exposure times (low millisecond to microsecond) do not permit the use of liquid jets for sample delivery, since they are too fast for useful diffraction data to be recorded from single crystals. Other methods originally developed for SFX have been used in SSX experiments, such as viscous extrusion (LCP and other matrices) (Botha *et al.*, 2015 ▸; Weinert *et al.*, 2019 ▸; Nogly *et al.*, 2015 ▸), fixed targets (Schulz *et al.*, 2018 ▸; Roedig *et al.*, 2016 ▸; Sherrell *et al.*, 2022 ▸) or hybrid methods such as conveyor belt-based methods (Beyerlein *et al.*, 2017 ▸). The advantages and disadvantages for each of these methods have been discussed extensively in the literature (Grünbein & Kovacs, 2019 ▸; Martiel *et al.*, 2019 ▸; Oberthuer *et al.*, 2017 ▸; Sierra *et al.*, 2018 ▸).

**Figure 1:**
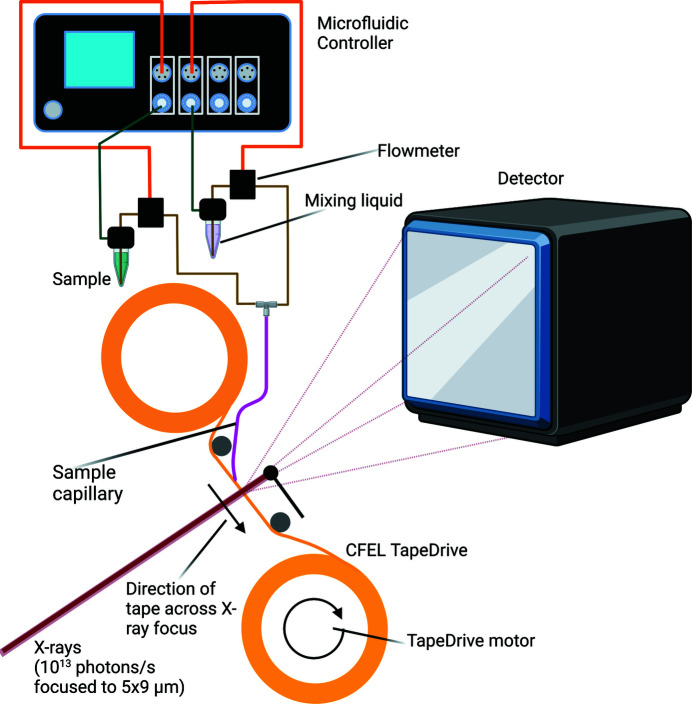
Schematic of the TapeDrive setup as used in this study. The sample capillary can be used with and without mixing, the installation remains the same. In this study we did not use the mix-and-diffuse method. The microfluidic controller is connected to the approximately 6 bar pressurized air available at PETRA III. The microfluidic controller and TapeDrive are controlled through a notebook that can be operated remotely from the hutch. TapeDrive is mounted on the motors that otherwise move the goniometer.

The CFEL TapeDrive (Fig. 1 ▸), developed at the Center for Free-electron Laser Science (CFEL) (Beyerlein *et al.*, 2017 ▸), offers the possibility of straightforward SSX experiments. Sample consumption is low (200–2000 nl min^−1^) and it is compatible with mix-and-diffuse methods for substrate or drug-design studies. Since it does not require modifications to the sample for delivery purposes, in the current setup, the sample vial (from batch crystallization) can be plugged directly into the liquid-dispensing system (Fig. 1 ▸) without the need for transfer, reducing the dead volume to about 5 µl (resulting from the necessary space between tubing and vial bottom to avoid self-clogging). Compared with viscous extrusion methods the tedious reservoir loading step is omitted and the alignment of the injector is not affected by sample change. Moreover, many datasets can be collected without the need to enter the experimental hutch (as is the case for all fixed-target systems to date), making the CFEL TapeDrive not only ready for further automation, but already now allowing for many datasets to be collected uninterrupted and in a very efficient way. This setup has been shown previously to enable efficient SSX experiments using lysozyme as a model system (Beyerlein *et al.*, 2017 ▸). We show here the versatility of this setup and methodology by performing structural studies at room temperature (RT) of three additional enzyme systems of biological relevance: (i) *Klebsiella pneumoniae* CTX-M-14 β-lactamase (Wiedorn *et al.*, 2018 ▸), a β-lactamase relevant to multi-antibiotic resistance mechanisms; (ii) *Nectria haematococca* xylanase GH11 (Andaleeb *et al.*, 2020 ▸), a highly active xylanase suitable for industrial applications; and (iii) *Aspergillus flavus* urate oxidase (UOX) in the presence of the long-lived intermediate-like 5-per­oxy-9-methyl-isourate (5PMUA). Using this system, data collection times and sample consumption are similar to that of traditional single-crystal MX.

## 2. Methods

### 2.1. Sample preparation

#### 2.1.1. CTX-M-14 β-lactamase

*K. pneumoniae* CTX-M-14 β-lactamase (CTX-M-14) was produced, purified and crystallized as described previously (Wiedorn *et al.*, 2018 ▸), with a slight modification in the crystallization conditions to obtain bigger crystals that roughly match the X-ray focal spot. For this purpose, a 50%(*v*/*v*) CTX-M-14 solution (22 mg ml^−1^) was mixed with a 45%(*v*/*v*) crystallizing agent [40%(*w*/*v*) PEG8000, 200 m*M* lithium sulfate, 100 m*M* sodium acetate, pH 4.5] and with a 5%(*v*/*v*) undiluted seed stock in batch crystallization setups. This resulted in crystals with a homogeneous size distribution of 11–15 µm after 90 min at 293 K. Crystals were centrifuged at 200*g* for 5 min and the supernatant was replaced with a stabilization buffer [28%(*w*/*v*) PEG8000, 140 m*M* lithium sulfate, 70 m*M* sodium acetate, pH 4.5] to prevent further crystal growth. Prior to the measurements, the microcrystal suspension was filtered using a 30 µm gravity flow filter (Celltrics, Sysmex).

#### 2.1.2. GH11 xylanase

*N. haematococca* GH11 xylanase (GH11) was produced and purified as described previously (Andaleeb *et al.*, 2020 ▸). For crystallization, the original conditions were modified to obtain microcrystals. Crystallization was carried out at 293 K. Initial crystals obtained from hanging drops under the crystallization conditions (1 *M* ammonium sulfate, 100 m*M* sodium citrate pH 5.5) were crushed under a stereomicroscope using a crystal crusher tool (Hampton research). The reservoir solution was pipetted to the drop and the seed stock was collected by washing the drop with reservoir solution. The seed stock was transferred to a seed bead tube (Molecular Dimensions Ltd, UK), vortexed three times for 30 s each, with an interval of 30 s between each vortex to obtain the final seed stock. Protein solution (15 mg ml^−1^), crystallizing agent and seed stock were mixed with a ratio of 1:1:0.5. The mixture was vortexed 4 times for 30 s in 10 min intervals. After 30 min, the microcrystals were centrifuged at 200*g* and the supernatant was replaced with a precipitant solution. By applying the same protocol, microcrystals were obtained under two crystallizing conditions: crystallizing agent 1:1 *M* (NH_4_)_2_SO_4_, 100 m*M* sodium citrate pH 5.5, and crystallizing agent 2:200 m*M* (NH_4_)_2_SO_4_, 100 m*M* sodium citrate pH 5.5 and 20%(*w*/*v*) PEG 6000. Microcrystals obtained under both precipitant conditions – with dimensions between 10 and 20 µm – were tested for diffraction data collection.

#### 2.1.3. Urate oxidase

Untagged *Aspergillus flavus* urate oxidase (UOX) was expressed in *E. coli* using a codon-optimized synthetic cDNA (Genscript, USA) inserted into a pET24b vector. Protein expression was performed in *E. coli* BL21(DE3) cells at 20°C for ∼20 h. Protein purification was achieved using a combination of ammonium sulfate precipitation, DEAE and RESOURCE Q ion-exchange, phenyl sepharose hydro­phobic interaction, and Superdex 75 size-exclusion chromatographic steps. For crystallization, UOX in Tris-acetate buffer (50 m*M*, pH 8.0) was concentrated to 20 mg ml^−1^ and saturated with 9-methyl uric acid (MUA). Large crystals were obtained at 20°C using the batch method by mixing the protein solution with an 8%(*w*/*v*) PEG 8000 reservoir in a 1:2 ratio under aerobic conditions. With this method UOX crystals typically reach dimensions of at least 400 × 400 × 300 µm. In the presence of O_2_ (air), MUA undergoes UOX-dependent di­oxy­genation, affording the mechanistically relevant 5PMUA peroxide derivative (Bui *et al.*, 2014 ▸). The latter is stable in the crystal for approximately 2–3 weeks. For the SSX experiment, a total of 12 large crystals of the UOX-5PMUA complex (approximate dimensions 800 × 400 × 400 µm) were vortexed in the presence of 1 mm diameter glass beads for about 10 min and then diluted in their mother liquor to yield 1 ml microcrystalline suspension with crystal sizes in the 3–20 µm range. Following fragmentation, we passed the microcrystalline slurry through a 30 µm mesh-size filter (CellTrics, Sysmex), resuspended and otherwise used as is.

### 2.2. Beamline setup

For sample delivery, we used a conveyor belt apparatus called TapeDrive (Fig. 1 ▸) (Beyerlein *et al.*, 2017 ▸). However, to minimize the dead volume and to make delivery more efficient, the sample was directly pressurized by the ElveFlow system. The sample vial (Eppendorf, Germany), in which the protein was crystallized (CTX-M-14 and GH11) and stored until use at either RT or 277 K (depending on crystal stability and time from crystallization to the SSX experiment), was connected to the Elveflow OB1 flow controller using a special adaptor available from ElveSys. A microfluidic flow sensor was also installed to control and monitor the flow rate. For mix-and-diffuse experiments (not used in this study) the setup is duplicated at a second channel at the Elveflow OB1 flow controller. The other side of the flow-meter was connected to a Kapton-coated borosilicate capillary with an internal diameter of 180 µm (Polymicro, USA), through which the microcrystal suspension flowed onto the tape. This capillary can easily be swapped out for a mixing dispenser as described previously (Beyerlein *et al.*, 2017 ▸). The end of the capillary that was in contact with the tape was sharpened for optimized sample flow onto the tape. The tape under the sample capillary was continuously moving, producing a sample stream on the tape aligned with the X-ray focus. As before (Beyerlein *et al.*, 2017 ▸), non-sticky polyimide tape with a width of 6 mm and a thickness of 12 µm (Caplinq, The Netherlands) was used. The tape position was vertically confined by grooves in the TapeDrive body that matched the width of the tape. The TapeDrive was mounted on the crystallography endstation at the P11 beamline at PETRA III (DESY, Hamburg) such that the 12.0 keV photon energy X-rays were focused at the sample position to a spot of 9 × 5 µm (width × height) with a maximum flux of about 1.6 × 10^13^ photons s^−1^. To control X-ray exposure to the crystals, a rotating beam chopper made of a 4 mm-thick brass plate with holes for the X-rays to pass through was placed upstream of the focusing optics. The signal from a photodiode placed downstream of the chopper was used to trigger the readout of a PILATUS 6M detector, resulting in the collection of one diffraction image per pulse. HiDRA was used to transfer data rapidly to the ASAP3 storage system at PETRA III and the incoming data stream was monitored with *OnDA* (Mariani *et al.*, 2016 ▸) for fast feedback during the experiment. The online hit-finding parameters were optimized to beamline setup and sample properties on-the-fly. Data collection parameters were directly controlled in the beamline controls system based on *TANGO Controls*. Data collection runs consisted of a maximum of 40 000 diffraction images. Data were collected at RT from randomly oriented microcrystals suspended in their crystallization buffer. For the first run of microcrystals of β-lactamase CTX-M-14 from *K. pneumoniae* the flow rate of sample was set to 2 µl min^−1^. In all other runs of CTX-M-14 and NhGH11 a stable sample flow rate of 1 µl min^−1^ was maintained. A tape speed of 1 mm s^−1^ was used throughout the experiment.

### 2.3. Data processing

Data processing, starting directly from the native CBF-files, was carried out using the *CrystFEL* package (version 0.8.0; White *et al.*, 2016 ▸, 2012 ▸). In *indexamajig*, the option --peaks = peakfinder8 was used to identify individual ‘hits’ from the complete set of collected diffraction patterns, as defined by an automatically generated list of files. Detected ‘hits’ were then indexed using *XGANDALF* (Gevorkov *et al.*, 2019 ▸), *XDS* (Kabsch, 2010 ▸), *mosflm* (Battye *et al.*, 2011 ▸), *asdf* and *DIRAX* (Duisenberg, 1992 ▸) (in that order), and integrated with the --int-radius = 3,4,8 option. The geometry input file was adapted for the photon energy and detector distance from previous experiments at P11. For CTX-M-14, the resulting unmerged streams of indexed and integrated diffraction data were then processed with *ambigator* (Brehm & Diederichs, 2014 ▸) in *CrystFEL* to resolve the indexing ambiguity. This was required as CTX-M-14 crystallizes in the enantio­meric *P*3_2_21 space group, and thus exhibits indexing ambiguities. Scaling and merging of the data were carried out for all samples in *partialator* in *CrystFEL* using three iterations and the --push-res = 2 option. For the 5k and the 10k datasets, partiality refinement using the xsphere model in *CrystFEL* was used. MTZ files for crystallographic data processing were generated from *CrystFEL* merged reflection datafiles using *F2MTZ* within the *CCP4* suite (Winn *et al.*, 2011 ▸). Figures of merit were calculated using *compare_hkl* (*R*_split_, CC_1/2_ and CC*) and *check_hkl* (SNR, multiplicity and completeness), which are both part of *CrystFEL*.

### 2.4. Structure solution and refinement

For CTX-M-14, the structure determined at the European XFEL (PDB entry 6gth; Wiedorn *et al.*, 2018 ▸) was used as the initial model (after removal of the ligand, avibactam). Initial refinement was carried out using *phenix.refine* (Adams *et al.*, 2010 ▸; Afonine *et al.*, 2012 ▸), with all isotropic atomic displacement parameters (ADPs) set to 20 Å^2^ and using simulated annealing. Ions and ordered solvent molecules were built into the model using *Coot*, TLS-groups were identified using the *TLSMD-server* (Painter & Merritt, 2006 ▸). Iterative cycles of restrained maximum-likelihood and TLS refinement using *phenix.refine* and manual model rebuilding using *Coot* (Emsley *et al.*, 2010 ▸) were carried out until convergence. *Polygon*, *MolProbity* (Chen *et al.*, 2010 ▸) and thorough manual inspection were used for validation of the final model.

For GH11, we processed data with *CrystFEL* as described above, with the addition that during the *partialator* step we used both the ‘unity’ and the ‘xsphere’ partiality models for comparative reasons. The structure of GH11 (PDB entry 6y0h; Andaleeb *et al.*, 2020 ▸) obtained under cryogenic conditions was used as the initial model and refinement was carried out essentially as described above, but without TLS-refinement. For the detailed investigation of the relation of data quality and length of data collection/indexed patterns, *phenix.refine* was used with exactly the same parameters and without manual intervention.

The RT SSX structure of the UOX–5PMUA complex was solved by Fourier difference methods starting from the model of the same complex obtained at near-atomic resolution under cryo-conditions and using low X-ray dose data (PDB entry 4cw2; Bui *et al.*, 2014 ▸). Prior to crystallographic refinement, the model was stripped of the bound ligand and all ordered solvent molecules. Crystallographic refinement was carried out using *Refmac5* of the *CCP*4 suite (Murshudov *et al.*, 2011 ▸; Steiner *et al.*, 2003 ▸). Restraints for the bound 5PMUA molecule were generated using a recent version of *AceDRG* (Long *et al.*, 2017 ▸) now available in the latest *CCP*4 release (version 8.0). *AceDRG* derives atom types from the Crystallography Open Database (Gražulis *et al.*, 2012 ▸).

*PyMol*, *ChimeraX* and *Coot* (Emsley *et al.*, 2010 ▸) were used for the generation of molecular images. For root-mean-square deviation (RMSD) calculations and alignment, *PyMol* and *ChimeraX* (Pettersen *et al.*, 2021 ▸) were used. Solvent channels and solvent channel maps for CTX-M-14 β-lactamase were calculated using *MAP_CHANNELS* (Juers & Ruffin, 2014 ▸).

## 3. Results

### 3.1. CTX-M-14

CTX-M-14 belongs to the extended spectrum β-lactamases (ESBLs) that play an important role in emerging multi-antibiotic resistance mechanisms (Perbandt *et al.*, 2022 ▸). This class of enzymes hydrolyze the β-lactam ring structure of most prominent antibacterial agents used in medicine and render them ineffective. The constantly evolving resistance to penicillin and penicillin-derived antibiotics is forcing the development of new antibiotics, as ESBLs in particular, including CTX-M-14 from *K. pneumoniae*, are already able to cleave antibiotics specifically developed against pathogens with high β-lactamase stability, including third-generation cephalo­sporins, such as cefotaxime or ceftazidime.

For CTX-M-14 we collected an initial SSX dataset of 10 000 diffraction images in 400 s (dataset 10k). We had originally planned a longer data collection but X-ray loss due to a beam dump at PETRA III prevented further progression. Nevertheless, this ‘mini’ dataset afforded a total of 4286 indexed images from only ∼13.3 µl of sample injected. This 42.9% ‘indexing fraction’ of the total collected detector exposures corresponds to an effective data collection rate of about 10.7 Hz with only 32.2 ng of protein consumed per indexable detector frame. Processing with the *CrystFEL* package indicated that these 4286 indexed frames already constitute a complete dataset to about 1.55 Å resolution with excellent statistics (Table 1 ▸). Crystallographic refinement indicators and the resulting electron density maps further confirmed the high quality of the data.

**Figure 2:**
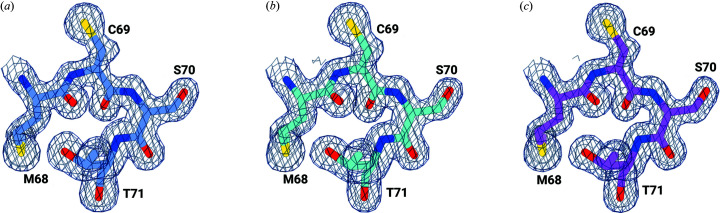
Selected residues of CTX-M-14 β-lactamase around the active center and overlayed with the corresponding 2*mF*_o_ − *F*_c_ maps contoured each at 1.5σ. (*a*) 10 000 detector images (10k), (*b*) 5000 detector images (5k), (*c*) full dataset (127 171 detector images). For (*a*) and (*b*) data out to 1.55 Å were used, for (*c*) the CC* statistics indicated that data out to 1.4 Å could be used. No significant differences between the electron density maps are visible.

Based on the serendipitous observation that a complete SSX dataset could be collected in less than 6 min, we carried out a more systematic investigation. Three more independent runs with microcrystals of CTX-M-14 were collected, yielding 61 331 indexable detector frames from a total of 127 171 recorded frames (dataset ‘all’). This corresponds to a 48.2% average ‘indexing fraction’ with 28.7 ng of protein consumed per indexable detector frame and a total volume of 91.4 µl microcrystalline suspension used. Data collection and refinement statistics are, as expected, superior for the combination of all runs, and the nominal resolution (based on a CC_1/2_ cut-off of 0.15) extends to 1.4 Å (in comparison with 1.55 Å for the ‘mini’ data collection). This improvement in statistics does not translate in obvious changes in electron density maps (see Fig. 2 ▸). We then looked at the first 5000 frames collected in the first run (dataset 5k). At 25 Hz (maximum frame rate of the PILATUS 6M) this corresponds to a data collection time of 200 s. From these 5000 images, 5109 crystals could be indexed with *CrystFEL* and data collection statistics show that with these 5109 indexed crystals, a full dataset to about 1.55 Å resolution can be obtained. This means that less than 3.5 min of data collection time is needed to obtain a full serial crystallography dataset to near-atomic resolution using monochromatic X-rays at a synchrotron. For these 5109 indexed crystals, a total of no more than 34 µg of protein in roughly 3.3 µl of microcrystalline suspension was used. This corresponds to only 6.65 ng of protein consumed per indexable detector frame. In comparison, 6250 ng per detector frame was used in the first SSX experiment (Stellato *et al.*, 2014 ▸) and 89 ng per detector frame for the lysozyme mix-and-diffuse study (Beyerlein *et al.*, 2017 ▸), a drastic reduction in sample consumption. Again, the refinement statistics and the resulting electron density maps are of excellent quality (see Table 1 ▸ and Fig. 2 ▸).

**Figure 3:**
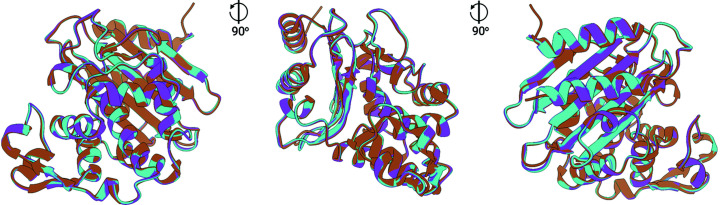
Comparison of the overall structures of CTX-M-14 β-lactamase from this study (cyan: 5k dataset; magenta: full dataset) and the cryoMX structure (PDB entry 7q0z, brown). It can be seen that, aside from the differences in symmetry and unit-cell dimensions, the asymmetric units align very well.

CTX-M-14 crystals used here have the same symmetry and unit-cell parameters as the microcrystals in complex with avibactam used at EuXFEL (Wiedorn *et al.*, 2018 ▸). They differ however from the larger crystals grown under similar conditions which are used for single-crystal MX under cryogenic conditions (Perbandt *et al.*, 2022 ▸). The datasets collected here allowed us to determine the first non-cryogenic structure of inhibitor-free CTX-M-14. Although the crystal contacts and symmetry are completely different from the corresponding cryogenic structure (PDB entry 7q0z; Perbandt *et al.*, 2022 ▸), the structures are very similar (RMSD of 0.332 Å, Figs. 3 ▸ and S1 of the supporting information), albeit a bit larger than the RMSD between the structures from different numbers of indexed crystals from this study (‘all’ versus 10k, 0.091 Å; ‘all’ versus 5k, 0.085 Å; 10k versus 5k, 0.093 Å). The RMSDs between the structure from EuXFEL (PDB entry 6gth; Wiedorn *et al.*, 2018 ▸) and the structures from this study lie in between and range from 0.161 Å (EuXFEL versus ‘all’) to 0.166 Å (versus 5k) and 0.169 Å (versus 10k). Like in the EuXFEL structure, two N-terminal residues that could be built in the cryogenic structure (E25 and T26) could not be built in all three RT-SSX structures due to the absence of interpretable electron density. On the other hand, an additional residue (L289) could be built into electron density in two of the three datasets (‘all’ and 10k) from this study, which is a residue that was absent in both the EuXFEL and the cryogenic structure. The different packing of the crystals in the cryogenic case and the microcrystal RT cases also results in a slightly different solvent accessibility within the crystal. Using the tool *MAP_CHANNELS* (Juers & Ruffin, 2014 ▸) we calculated solvent channels within the crystals and indeed the channels for the microcrystals at RT are slightly larger than those in the larger crystals used for cryoMX, making mix-and-diffuse studies theoretically more feasible (see Fig. S2). The *B* factors and Wilson *B* factors of all RT structures are very similar and, as expected, higher than those of the structure refined against data collected under cryogenic conditions.

### 3.2. GH11

**Figure 4:**
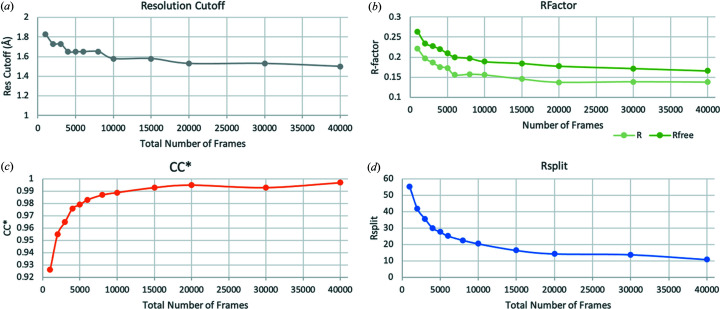
Evolution of crystallographic and refinement metrics for NhGH11 as a function of the number of detector frames included in a dataset. (*a*) Evolution of resolution cut-off (resolution at which CC* > 0.5) as a function of addition of further detector frames. (*b*) Evolution *R*_work_ and *R*_free_ values using the same starting model and refinement parameters and high-resolution cut-off of 1.9 Å. (*c*) Overall CC* and (*d*) overall *R*_split_ values as a function of detector frames added, calculated to a high-resolution cut-off of 1.8 Å.

**Figure 5:**
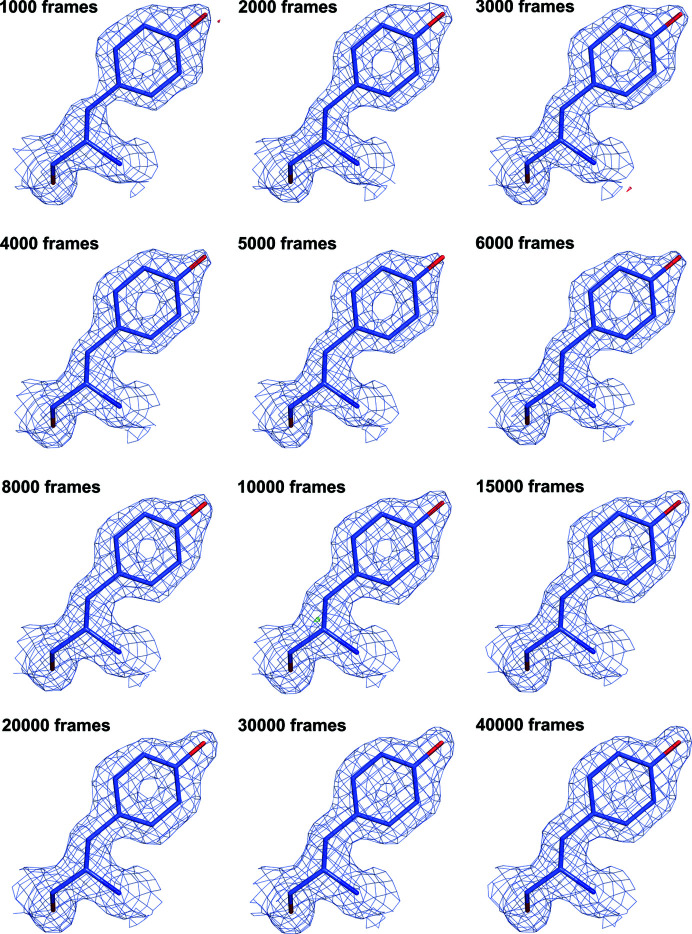
σ-weighted 2*F*_o_ − *F*_c_ electron density around Tyr41 of NhGH11 xylanase for models refined against the datasets made using the stated number of frames. Resolution cut-off for all datasets was 1.9 Å and all maps are displayed at 1σ.

**Figure 6:**
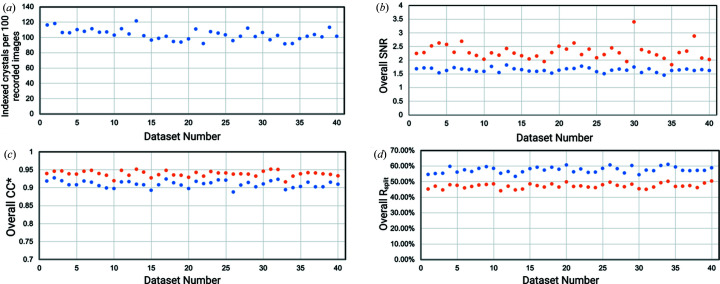
Crystallographic metrics and indexing rates for all 40 NhGH11 datasets. (*a*) Indexing rate, (*b*) SNR, (*c*) CC* and (*d*) *R*_split_ values for all 40 datasets, each consisting of 1000 recorded detector frames within one run. In (*b*), (*c*) and (*d*), values from processing without partiality modeling are depicted in blue and values from processing with partiality modeling are depicted in orange.

For GH11, a single run consisting of 40 000 detector images was collected in 26 min and 40 s. To inspect fluctuations in data quality and indexing rates, the run was split into 40 independent datasets, each consisting of 1000 detector images, and processed with the CrystFEL pipeline separately. Next, 1000, 2000, 3000, 4000, 5000, 6000, 8000, 10 000, 15 000, 20 000, 30 000 and finally all 40 000 detector images were processed together with partialator to assess the number of indexed images needed for a full dataset. Going from 1000 images to 40 000 images, the resolution at which CC* reaches 0.5 (the typical cut-off level in macromolecular crystallography) goes from 1.83 Å down to 1.5 Å [see Fig. 4 ▸(*a*)]. Using the same parameters in *phenix.refine* and a high-resolution cut-off at 1.9 Å, *R*_free_ values drop from 0.264 (1000 images) to 0.166 (40 000 images). The improvement in reasonable resolution and *R*_free_ with the addition of more images merged, however, is not uniform, as can be seen in Fig. 4 ▸(*b*). *R*_free_ drops from 0.264 to 0.220, 0.209 and 0.189 when merging 4000, 5000 and 10 000, respectively, instead of 1000 detector images and only drops further to 0.166 for the full run (40 000 images). Similarly, the resolution at which CC* of the highest resolution shell reaches 0.5 drops from 1.83 to 1.65 Å using 4000 instead of 1000 images, and only drops to 1.58 Å at 10 000 images and 1.50 Å at 40 000 images [Fig. 4 ▸(*a*)]. Similar progress can be observed when looking at overall values of CC* or *R*_split_ [Figs. 4 ▸(*c*) and 4(*d*)]. Improvement in data quality can be best assessed when looking at the evolution of electron density maps with the addition of more indexed detector frames (Fig. 5 ▸). Here maps generated from automatic refinement [uniform resolution cut-off at 1.9 Å, same input mode, same parameters, see Fig. 4 ▸(*b*) for corresponding *R*_work_ and *R*_free_] around Tyr41 are shown and clear improvement of map quality with the addition of more detector images can be observed. When looking at the 40 datasets, each consisting of 1000 detector images, within these datasets there is no clear trend, not with the number of indexable patterns per frame, the crystallographic figures of merit (SNR, *R*_split_, CC_1/2_ or CC*, Fig. 6 ▸), nor for the *R*_free_ values after automatic refinement (Fig. S4). The indexing rate is very high and ranges from 91.5 to 121.5% and *R*_free_ values from 0.259 to 0.306, and the number of indexable frames and resulting *R*_free_ values are not clearly correlated (Figs. 6 ▸ and S4).

Partiality refinement in CrystFEL partialator with the xsphere partiality model was also used to assess whether improvements could be observed, especially for the case using only 1000 images per dataset. In all 40 individual 1000-image datasets the data quality metrics (*R*_split_, CC*, CC_1/2_, SNR) improved when using partiality refinement [Figs. 6 ▸(*b*)–6(*d*)]. In most cases the quality improvement was quite uniform across the 40 datasets, *i.e.* similar relative changes upon partiality refinement. Over all 40 datasets CC* increased by 0.029 ± 0.007 (from 0.910 ± 0.009 to 0.939 ± 0.008). However, there were some outliers, for example dataset 30, where overall SNR improved from 1.74 to 3.40, and overall CC_1/2_ changed from 0.71 to 0.81. Using automated refinement with the same conditions across all datasets for both conditions (with and without partiality modeling), the improvement through partiality refinement was less clear as indicated by the crystallographic metrics (Fig. S4). In some cases, *R*_work_ and *R*_free_ did not improve for the same dataset after partiality modeling in CrystFEL; however, this was only true for 15% of cases (six in total). In one case *R*_free_ remained the same and in 82.5% there were slight improvements through partiality modeling. The average *R*_free_ over all 40 datasets dropped by 0.006 ± 0.001 (from 0.281 ± 0.002 to 0.275 ± 0.002) and the average *R*_work_ dropped by 0.004 ± 0.001 (from 0.243 ± 0.002 to 0.239 ± 0.001).

The high and relatively uniform indexing rate and the absence of a trend in crystallographic or refinement statistics implies that, for these crystals at least, settling or other negative effects over the duration of one run cannot be observed and that TapeDrive can be used for stable and carefree data collection.

Additionally, we observed that, for the CTX-M-14 β-lactam­ase, the Wilson *B* factor and the ADP from the refinement appear independent of the number of indexed crystals in a dataset. With the GH11 individual datasets, we could extend this investigation to the range from 1000 to 40 000 detector images. Using the same refinement strategy and input PDB, the average isotropic ADP drops slightly from 23.4 (1000 images) to 22.8 (40 000 images); however, again no clear trend is visible, as the ADP is 23.4 with 30 000 detector images (Fig. S3).

**Figure 7:**
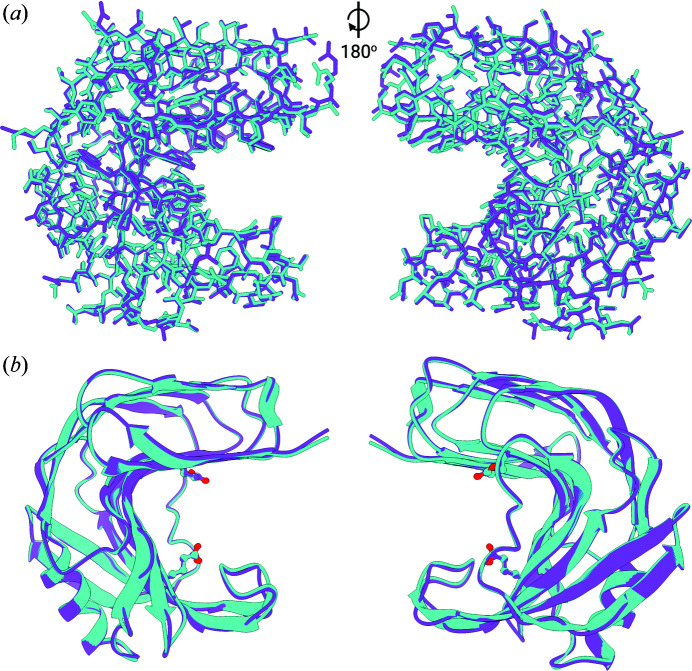
Comparison of the overall structures of NhGH11 xylanase obtained by cryoMX (cyan) and RT-SSX (magenta, 40 000-image dataset). (*a*) All-atom ‘stick’ depiction of the proteins, allowing us to assess the alignment of the sidechains. (*b*) Cartoon-plot for better visibility of the overall fold and and residues E89 and E180 that form the catalytic center. These residues overlap almost perfectly for both datasets.

**Figure 8:**
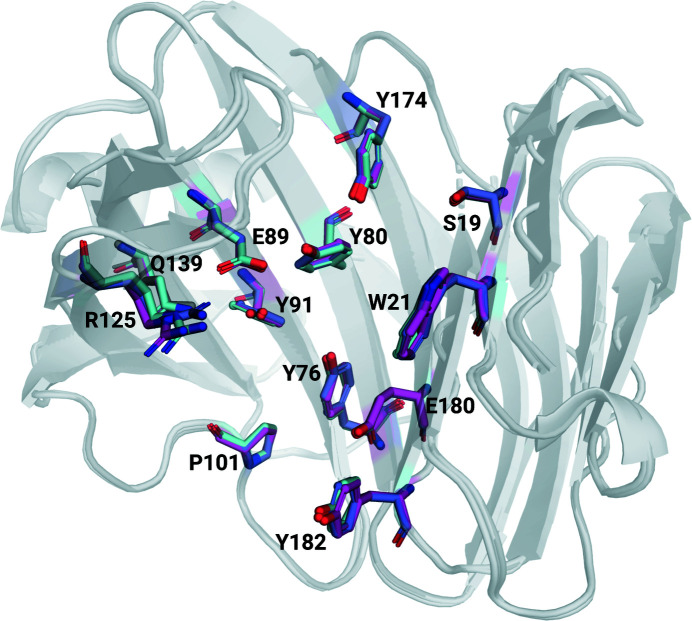
Detailed view of the alignment of all residues composing the active site of NhGH11 xylanase. The cryoMX residues are shown in cyan, the full 40 000-image dataset structure are shown in magenta and additionally the active site residues from the structure obtained by refinement against the 1000-image dataset are shown in purple. The strongest deviations from the cryoMX reference can be observed in Arg125, all other residues overlap almost perfectly.

In the case of *N. haematococca* xylanase (GH11) the crystal symmetry does not change with crystal size. The crystals used for cryoMX (PDB entry 6y0h; Andaleeb *et al.*, 2020 ▸) are almost isomorphous to those used in this RT-SSX study, with the latter ones having slightly larger unit cells [*a* = 80.55 Å, *b* = 38.85 Å, *c* = 53.57 Å, α = 90.00°, β = 91.00°, γ = 90.00° (RT) versus *a* = 79.45 Å, *b* = 38.50 Å, *c* = 53.59 Å, α = 90.00°, β = 91.43°, γ = 90.00° (cryo)]. The structural models resulting from refinement against the RT-SSX datasets are the first RT datasets of this *N. haematococca* xylanase. The structures superimpose very well with the structure from cryoMX (C_α_ RMSDs ranging from 0.226 Å for the 1000-image dataset to 0.213 Å for the 40 000-image dataset, Fig. 7 ▸) and differences are mostly visible in the orientations of side chains. The same number of residues could be built into electron density in all RT-SSX datasets and there are no differences to the cryoMX structure. The two catalytic residues E89 and E180 entirely overlap, with an all-atom RMSD of 0.126 Å (Fig. 7 ▸). The alignment of all the residues of the active site (S19, W21, Y76, Y80, E89, Y91, P101, R125, Q139, Y174, E180 and Y182) is slightly worse, with an all-atom RMSD of 0.519 Å and comparable with the all-atom RMSD of all the residues (0.583 Å). The same alignment carried out using the 1000-image dataset, instead of the 40k dataset, resulted in a slightly worse all-atom RMSD of 0.630 Å (Fig. 8 ▸). For just the two catalytic residues E89 and E180, the all-atom RMSD is 0.151 Å, overall the residues in the active site align very well in all three cases (Fig. S5). This shows that (*a*) the addition of more images in an SSX dataset increases – as expected – the accuracy of the resulting model; but also (*b*) that the improvements, even with a 40 times longer data collection time, are rather minor. This shows that useful models, even those accurate enough for mechanistic studies, can be generated from the refinement of datasets of just 1000 detector images, corresponding to only 40 s data collection time.

### 3.3. UOX-5PMUA complex

Unlike the other experiments discussed here, in the case of the UOX-5PMUA complex no attempt was made to grow microcrystals. Instead, we purposefully crushed twelve large crystals (approximate volume of each crystal 0.13 mm^3^) using 1 mm-diameter glass beads to explore whether this rather coarse approach could be useful for SSX experiments, for example, when microcrystal optimization is problematic. We did not experiment with bead size but considered 1 mm beads a suitable choice as a previous report that used fragmentation for transmission electron microscopy analysis indicated that the use of 0.5 and 1.0 mm beads resulted in homogeneous populations of crystal fragments of low-micrometre sizes (Stevenson *et al.*, 2016 ▸). The same study found that the standard 3.0 mm bead yielded inhomogeneous fragmentation with large crystal sizes still present in the solution whereas smaller beads (0.1 mm) produced no UV-detectable crystals. Using the fragmentation method, we collected a total of 170 905 frames with 3142 patterns indexed successfully (total data collection time was 6836 s or 114 min). This low percentage (1.84%) is due to the high dilution of the crystal slurry that resulted in many empty frames. The number of indexed patterns matched the number of crystals, indicating that all microcrystals were successfully indexed and that there were no ‘multiple hits’. Overall, this approach afforded, after merging, a complete 2.3 Å resolution dataset that was employed to determine the structure of the UOX-5PMUA complex at RT. Data collection and refinement statistics are reported in Table 1 ▸.

**Figure 9:**
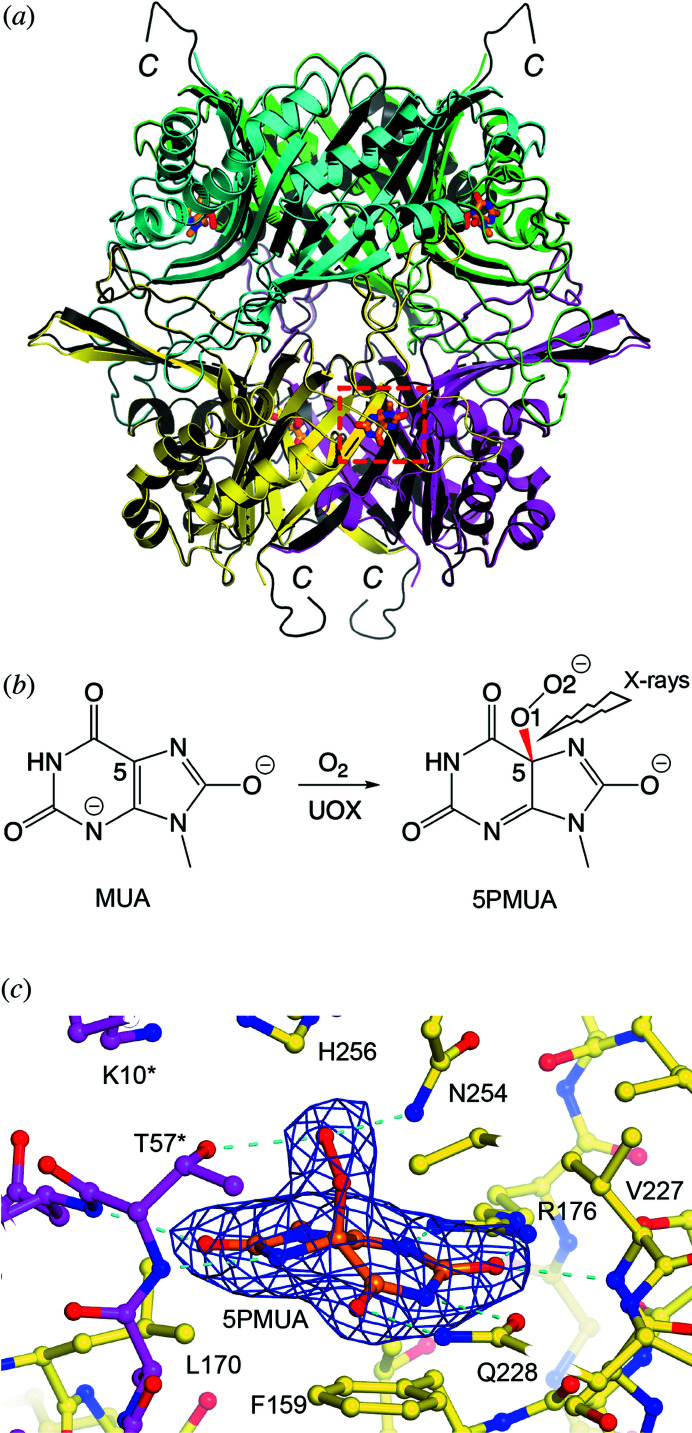
(*a*) Comparison of the UOX-5PMUA complex at RT and 100 K. The four UOX protomers from the RT structure are shown in yellow, magenta, green and cyan while the whole UOX tetramer (PDB code 4cw2) solved at 100 K is shown in black. The four 5PMUA molecules that bind at the interface between two protomers are shown as sticks; one is highlighted by a dashed red box for clarity. The flexible C-terminus (indicated by the letter C) is not observed in the present RT structure. (*b*) Scheme of the reaction that converts UOX-bound MUA into its 5PMUA derivative under aerobic conditions. The C5—O1 bond that is very susceptible to X-ray-induced rupture is shown in red. (*c*) Active site of the UOX-5PMUA complex from SSX data at 2.3 Å resolution. UOX residues at the interface are shown in stick representation and are color-coded according to the subunit they belong to. 2*mF*_o_ − *DF*_c_ electron density contoured at the 1σ level is shown in blue for 5PMUA. Hydrogen bonds involving 5PMUA are shown as cyan dashed lines.

UOX crystallizes in space group *I*222 with one monomer in the crystallographic atomic unit. The tetrameric arrangement with the *D*_2_ point group that constitutes its quaternary structure is generated in the crystal by symmetry. The active site is located at the interface between two protomers with the UOX tetramer able to bind four ligands. The UOX-5PMUA complex was previously solved at near-atomic resolution under cryo-conditions (100 K) (PDB entry 4cw2). We find that cell dimensions are modestly affected by cryo-cooling with a unit-cell volume contraction of about 2.1% (*a* = 79.79/79.51 Å, *b* = 96.03/95.13 Å, *c* = 105.21/104.31 Å – RT/100 K). Overall, the present RT medium-resolution complex is virtually identical to that at 100 K and near-atomic resolution with an RMSD of 0.23 Å for all common main-chain atoms in the residue range (1–295) [Fig. 9 ▸(*a*)]. The C-terminal region (296–301) is flexible and not visible in our RT structure although this is often the case even at cryo-conditions when the resolution is not as high as near-atomic.

One of the strengths of serial crystallography approaches is its reduced impact on radiation-sensitive samples. This is particularly relevant at RT where the absorbed dose that induces global radiation damage can be two orders of magnitude lower than those at cryogenic temperatures (Nave & Garman, 2005 ▸; Southworth-Davies *et al.*, 2007 ▸). Previous work has shown that the C5—O1 bond of 5PMUA [highlighted in red in Fig. 9 ▸(*b*)] is very susceptible to radiolysis. At 100 K, an average diffraction-weighted dose (DWD) of about 200 kGy results in the complete rupture of the C5—O1 bond and values as low as 2.5 kGy are required to minimize this (Bui *et al.*, 2014 ▸; Bui & Steiner, 2016 ▸). Specific radiation damage in 5PMUA manifests itself with the development of negative density peaks in difference Fourier maps along the C5—O1 bond reflecting its rupture while positive density appears in the heterocyclic plane because of the loss of pyramidalization at C5 with this carbon atom transitioning from *sp* ^3^ to *sp* ^2^ hybridization. Concomitantly, di­oxy­gen is liberated from the peroxide moiety and, at cryo-temperatures, remains trapped above it (Bui *et al.*, 2014 ▸; Bui & Steiner, 2016 ▸).

The present SSX experiment offered us the opportunity to study the UOX-5PMUA complex at RT. Already at the initial stages of refinement, difference Fourier maps revealed unambiguously that MUA reacted with O_2_ to produce the 5PMUA adduct in the crystal [Fig. S6(*a*)]. As previously observed in the structure of the complex solved at cryo-temperature, 5PMUA binds at the interface between two protomers of the UOX tetrameric assembly in a cavity lined by K10*, I54*, A56*, T57*, D58*, F159, R176, L170, S226, V227, Q228, N254, H256, G286, I288 (the asterisk indicates residues belonging to a separate UOX chain) with the peroxide moiety sandwiched between the side chains of T57* and N254. Inspection of electron density maps obtained from the SSX experiment after refinement does not reveal obvious signs of 5PMUA radiolysis [Fig. 9 ▸(*c*)]. The peroxide moiety displays pronounced pyramidalization at C5 accompanied by the strong distortion of its fused heterocycles. The C5—O1 bond length refines at the target value of 1.44 Å with no indication of C5—O1 rupture visible in *mF*_o_ − *DF*_c_ maps at the ±3σ level. We observe some negative density near the C5—O1 bond at the ±2.5σ level [Fig. S6(*b*)], however, this is matched by similar peaks in other regions of the maps thus suggesting that noise is a contributing factor at this threshold. Exact dose calculations are not straightforward for the present TapeDrive experiment because of complicating factors such as non-homogenous crystal size and variable crystal positioning with respect to the Gaussian-shaped beam. Nonetheless, DWD estimation using *RADDOSE-3D* (Zeldin *et al.*, 2013 ▸; Bury *et al.*, 2018 ▸) provides a value of approximately 70 kGy as an upper limit. The *RADDOSE-3D* script used for the calculation is available in the supporting information.

Overall, the quick ‘crush-and-collect’ approach employed here allowed the determination of the RT structure of the UOX-5PMUA complex and the visualization of the radiation-sensitive 5PMUA intermediate at medium resolution.

## 4. Discussion

Serial crystallography has made tremendous progress in the past five years, especially for its use at synchrotron light sources. It has brought back data collection at RT and revived time-resolved approaches to track structural dynamics of biological macromolecules. The conveyor belt based Tape­Drive combines – as a hybrid approach – the advantages of fixed-target and liquid-jet based sample delivery. Here, we showed that sample consumption per dataset using this system is *en par* or even lower than with fixed-target (Roedig *et al.*, 2017 ▸; Schulz *et al.*, 2018 ▸) or viscous-extrusion approaches (Botha *et al.*, 2018 ▸) and benefits directly from optimization of crystallization to achieve a high yield of homogenous microcrystals. At the same time, the TapeDrive system allows for uninterrupted data collection without manual intervention or the need to enter the hutch, enabling future automated multi-parameter data collection for multidimensional serial crystallography (Mehrabi *et al.*, 2021 ▸). This was demonstrated by collecting 40 datasets in less than 30 min, wherein each dataset had good statistics and usable diffraction to a resolution of better than 2 Å and each dataset took 40 s to collect with a PILATUS 6M detector, corresponding to only 7.5 s using an EIGER 16M (Dectris, Switzerland) at 133 Hz. More notably, in this setting less than 5 µg of protein was used per dataset, which would drop to below 1 µg of protein per dataset when using the EIGER. If a higher resolution is desired, longer data collection times improve resolution, *i.e.* recording more indexable detector images, with a sweet spot, in this case, at roughly 4000 images. However, looking at the resulting models and the accuracy of the positions of the active side-residues of NhGH11 (Fig. 8 ▸), the differences between the datasets from 40 000 to 1000 recorded detector images are rather marginal and the 40 times longer data collection time and 40 times higher sample consumption are not justified by the improvement. For further improvement, without using more sample or longer data collection times, the partiality refinement option in CrystFEL can be used which, for small datasets, does not slow down data processing. In this study it improved crystallographic statistics across all 40 datasets, even though the effect on the refinement statistics was not entirely uniform and its use has to be assessed on a case-to-case basis.

SSX experiments benefit from homogenous micro-crystals. Strategies have been proposed to reproducibly achieve this by modifying the conditions used to produce single well ordered crystals that have been traditionally the focus of many crystallographic laboratories (Tenboer *et al.*, 2014 ▸; Beale *et al.*, 2019 ▸; Stohrer *et al.*, 2021 ▸). Although the correct understanding of the crystallization phase diagram is undoubtedly beneficial to facilitate the macrocrystal-to-microcrystal transition, the complex nature of biological crystal formation suggests that this can be a time-consuming process. Fragmentation of large, imperfect crystals into microcrystals or nanocrystals has been used for the structure determination of various test proteins by the electron cryo-microscopy method of microelectron diffraction (microED) (de la Cruz *et al.*, 2017 ▸). Here, we employed the rather crude approach of crushing large crystals for SSX. In the case of the UOX-5PMUA complex, this method still obtained a complete dataset at 2.3 Å resolution with satisfactory diffraction statistics. At present, we are unable to comment on how the quality of these crushed microcrystals compares with those ‘grown-for-purpose’, although the latter will provide a more homogenous population that can be beneficial for mix-and-diffuse studies. Nonetheless, our experiment with UOX-5PMUA shows that should micro-crystal optimization be problematic, this is not necessarily a roadblock and crushing standard-size crystals can be attempted if enough are available. It is expected that the success of this approach and the resolution attained will be very much dependent on the quality of the original crystals.

By their nature, serial methods distribute the absorbed energy over a multitude of crystals, instead of accumulating it as in oscillation-based data collections. This is particularly important at RT where, compared with cryo conditions, radiation damage degrades data quality at much lower doses. Therefore, though not providing completely radiation-damage-free data, serial approaches are able to mitigate the negative impact of radiolysis in macromolecular crystallography (de la Mora *et al.*, 2020 ▸; Nass, 2019 ▸). In addition to global effects, loss of diffraction power being the most obvious, radiation damage also leads to specific changes in biological molecules. De­carboxyl­ation of acidic residues, rupture of di­sulfide bridges and geometric alterations at metal centers due to photoreduction are rather common effects (Weik *et al.*, 2000 ▸; Burmeister, 2000 ▸; Ravelli & McSweeney, 2000 ▸; Yano *et al.*, 2005 ▸; Ebrahim *et al.*, 2019 ▸).

In the case of the UOX-5PMUA complex, radiolysis of the bound ligand leads to C5—O1 bond breakage with concomitant flattening of the fused ring system and liberation of O_2_ emanating from the peroxo moiety (Bui *et al.*, 2014 ▸). Under cryo-conditions using the oscillation method, DWD in the 50–100 kGy range are sufficient to produce clearly visible damage (Bui *et al.*, 2014 ▸; Bui & Steiner, 2016 ▸). We were therefore surprised that the present SSX experiment did not highlight obvious radiation-damage effects on PMUA considering that DWD was on the order of tens of kilograys with an estimated upper limit of around 70 kGy. Although limited resolution and data quality as well as non-isomorphism induced by global radiation damage will undoubtedly contribute to swamping peaks in Fourier difference maps, thus concealing some specific damage, it appears unlikely that these effects can mask C5—O1 bond rupture completely, and our dose is still well below the rough limit of 380 kGy suggested to minimize global damage (de la Mora *et al.*, 2020 ▸). Previous studies have highlighted differences in radiation damage effects at room- and cryo-temperature with respect to S—S bond breakage and de­carboxyl­ation of acidic residues (de la Mora *et al.*, 2020 ▸; Gotthard *et al.*, 2019 ▸). A particularly intriguing observation is that for lysozyme crystals the Fourier difference maps at RT did not show damage at carboxyl groups of Glu and Asp residues, in contrast to what is observed in cryo-control series (de la Mora *et al.*, 2020 ▸). Although various hypotheses can be put forward with respect to the apparent lack of damage of 5PMUA in the current SSX experiment, including radical chemistry that facilitates recombination or the possibility of a dose-rate effect, further systematic investigations will be required to explore this in detail. Considering that crystallographic experiments at near-physiological temperatures are becoming more common, a deeper understanding of radiation damage under these conditions, particularly at specific sites, is of the utmost importance.

In summary, TapeDrive allowed for seamless, rapid, efficient and radiation-damage-free data collection at synchrotron light sources. In this study the frame rate of the PILATUS 6M detector was the bottleneck for efficiency and the use of high frame rate detectors – like the EIGER 2XE, LAMBDA or JUNGFRAU – will further improve this method and its usability as a standard tool for MX data collection, with data collection times and sample consumption per dataset reaching those for cryoMX at highly automated state-of-the-art beamlines. Having optimized samples, as was the case for NhGH11 and CTX-M-14 β-lactamase (an effort comparable to the optimization of crystals for cryoMX), between 500 and 1000 datasets could, in principle, be collected within 12 h. Since one tape roll lasts for more than 12 h, one can collect these datasets without ever entering the hutch, and perform all experiments that are fully automated and even remote. Using an EIGER 16M at a frame rate of 133 Hz, instead of the PILATUS, up to 5400 datasets in 12 h are possible. Alternatively, if one wanted to extend the size of a dataset to 4000 detector images to more safely ensure a complete dataset, still more than 1000 datasets could be collected in 12 h, which is slightly more than the number of datasets possible at high-throughput cryoMX beamlines, and certainly more than what is currently possible for SSX with fixed targets. With this and further developments already under way, TapeDrive will enable autonomous and high-output data collection, from drug screening at physiological pH values and temperatures, to combined temperature-, concentration-, time- and pH-resolved 7D multidimensional investigations.

## Supplementary Material

Figs. S1-S6, RADDOSE-script and tables of data processing information. DOI: 10.1107/S2052252522010193/zf5019sup1.pdf

PDB reference: RT SSX structures:CTX-M-14, 7zpv

PDB reference: CTX-M-14 (10k dataset), 8af7

PDB reference: *K. pneumoniae* CTX-M-14 (5k dataset), 8af8

PDB reference: *N. haematococca* GH11 xylanase (40k dataset), 8af4

PDB reference: GH11 xylanase (10k dataset), 8af5

PDB reference: GH11 xylanase (4k dataset), 8af6

PDB reference: GH11 xylanase (1k dataset), 7zq0

PDB reference: cofactor-free *A. flavus* urate oxidase complex, 7qar

## Figures and Tables

**Table 1:** Data collection and refinement statistics Values in parantheses are for the highest-resolution shell.

	CTX-M-14 β-lactamase ‘all’	CTX-M-14 β-lactamase (approximately 10000 frames)	CTX-M-14 β-lactamase (5000 frames)	NhGH11 xylanase (40000 frames)	NhGH11 xylanase (10000 frames)	NhGH11 xylanase (4000 frames)	NhGH11 xylanase (1000 frames)	UOX-5PMUA complex
PDB entry	7zpv	8af7	8af8	8af4	8af5	8af6	7zq0	7qar
Temperature (K)	293	293	293	293	293	293	293	293
No. of images collected	127170	10382	5000	40000	10000	4000	1000	170905
Total measuring time (s)	5086	415.3	200	1600	400	160	40	6386
Average acquisition rate (frames s^−1^)	25	25	25	25	25	25	25	25
Exposure time (ms)	7.48	7.48	7.48	3.78	3.78	3.78	3.78	3.78
Indexed lattices	61331	4286	5109	41508	10807	4402	1161	3142
Indexed per second	12.1	10.3	25.6	25.9	27.0	27.5	29.0	0.5
Space group	*P*3_2_21	*P*3_2_21	*P*3_2_21	*C*121	*C*121	*C*121	*C*121	*I*222
*a*, *b*, *c* (Å)	42.16, 42.16, 234.35	42.16, 42.16, 234.35	42.16, 42.16, 234.35	80.55, 38.85, 53.57	80.55, 38.85, 53.57	80.55, 38.85, 53.57	80.55, 38.85, 53.57	79.79, 96.03, 105.21
α, β, γ (°)	90, 90, 120	90, 90, 120	90, 90, 120	90, 91.00, 90	90, 91.00, 90	90, 91.00, 90	90, 91.00, 90	90, 90, 90
Solvent content	0.397	0.397	0.397	0.376	0.376	0.376	0.376	0.586
Resolution (Å)	1.40–17.77 (1.40–1.42)	1.55–17.77 (1.55–1.58)	1.55–17.77 (1.55–1.58)	1.51–17.16 (1.51–1.54)	1.63–17.50 (1.63–1.66)	1.70–17.16 (1.70–1.73)	1.90–16.92 (1.90–1.93)	2.30–17.07 (2.30–2.34)
Unique reflections	49225 (4733)	36504 (3514)	36495 (3514)	25916 (2291)	20896 (2091)	18431 (1813)	13235 (1325)	18265 (1796)
〈*I*/σ(*I*)〉	9.11 (0.69)	3.25 (0.62)	3.75 (0.63)	5.83 (0.55)	3.67 (0.66)	2.64 (0.47)	1.91 (0.68)	2.44 (0.52)
Completeness (%)	100 (100)	99.98 (100)	99.98 (100)	98.75 (87.48)	100 (100)	100 (100)	99.73 (99.33)	99.98 (100)
Multiplicity	2217 (1400)	114 (65.0)	149 (75.1)	432 (43.8)	134 (53.5)	59 (28.1)	20.98 (13.2)	28.20 (20.6)
*R*_split_	7.2 (165.91)	27.8 (172.41)	22.97 (170.30)	13.42 (248.68)	22.55 (168.38)	31.81 (245.34)	52.40 (169.81)	36.68 (193.69)
CC_1/2_	0.991 (0.266)	0.815 (0.268)	0.907 (0.262)	0.987 (0.152)	0.960 (0.189)	0.910 (0.158)	0.751 (0.185)	0.849 (0.225)
CC*	0.998 (0.648)	0.948 (0.650)	0.975 (0.644)	0.997 (0.514)	0.990 (0.564)	0.976 (0.522)	0.926 (0.559)	0.958 (0.606)
Wilson *B* factor (Å^2^)	24.22	23.58	23.14	19.51	20.59	21.11	18.68	27.31
Resolution range in refinement (Å)	1.40–17.78 (1.40–1.47)	1.55–16.54 (1.55–1.605)	1.55–14.95 (1.55–1.605)	1.51–16.92 (1.51–1.57)	1.63–16.92 (1.63–1.69)	1.70–16.92 (1.7–1.761)	1.90–16.92 (1.90–2.00)	2.30–17.08 (2.30–2.36)
Reflections used in refinement	49225 (4832)	36504 (3523)	36495 (3521)	25571 (2028)	20858 (2067)	18305 (1704)	13227 (1286)	17361 (1274)
Reflections used for *R*_free_	1033 (100)	768 (74)	765 (73)	1985 (156)	1618 (159)	1415 (128)	1021 (95)	919 (60)
*R*_work_	0.1410 (0.3338)	0.1797 (0.3015)	0.1758 (0.3775)	0.1516 (0.3561)	0.1625 (0.3257)	0.1890 (0.3535)	0.2272 (0.3083)	0.2182 (0.366)
*R*_free_	0.1561 (0.3827)	0.2147 (0.3192)	0.1988 (0.3643)	0.1831 (0.3590)	0.2104 (0.3683)	0.2203 (0.3787)	0.2617 (0.3571)	0.2661 (0.348)
RMS (bonds)	0.009	0.006	0.011	0.009	0.010	0.008	0.004	0.008
RMS (angles)	1.01	0.82	1.18	1.06	1.07	0.72	0.626	1.388
Ramachandran favored (%)	97.68	97.27	98.06	96.26	96.26	96.26	95.72	96.92
Ramachandran allowed (%)	1.93	2.34	1.55	3.21	3.74	3.74	4.28	3.08
Ramachandran outliers (%)	0.39	0.39	0.39	0.53	0.00	0.00	0.00	0.00
Rotamer outliers (%)	1.87	1.83	0.95	2.86	2.86	2.92	2.34	0.00
Clashscore	2.19	6.90	3.47	5.17	5.17	3.61	9.24	0.62
Average *B* factor	34.26	32.61	34.56	23.58	23.57	24.78	23.74	39.13
Macromolecules	33.23	31.50	32.66	21.32	21.23	22.53	22.44	39.40
Solvent	45.06	44.97	123.90	39.88	39.55	37.22	32.03	34.69
Ligands	78.08	45.56	46.35	–	–	–	–	33.48
*MolProbity* Score	1.27	1.27	0.89	1.85	1.83	1.79	2.07	0.87
