# Supplementary material for: Rapid and efficient room-temperature serial synchrotron crystallography using the CFEL TapeDrive
Source: IUCrJ. 2022 Oct 31;9(Pt 6):778–91. doi: 10.1107/S2052252522010193 (PMC9634612; doi:10.1107/S2052252522010193)
Supplement: Supplementary file 1 [file m-09-00778-sup1.pdf]

IUCrJ

Volume 10 (2023)

Supporting information for article:

Rapid and efficient room-temperature serial synchrotron crystallography using the CFEL TapeDrive

Kara A Zielinski, Andreas Prester, Hina Andaleeb, Soi Bui, Oleksandr Yefanov, Lucrezia Catapano, Alessandra Henkel, Max O. Wiedorn, Olga Lorbeer, Eva Crosas, Jan Meyer, Valerio Mariani, Martin Domaracky, Thomas A. White, Holger Fleckenstein, Iosifina Sarrou, Nadine Werner, Christian Betzel, Holger Rohde, Martin Aepfelbacher, Henry N. Chapman, Markus Perbandt, Roberto A. Steiner and Dominik Oberthür

Rapid and efficient room temperature serial synchrotron crystallography using the CFEL TapeDrive

Kara A. Zielinski[1,*,$], Andreas Prester[2,*], Hina Andaleeb[3,*,$$], Soi Bui[4], Oleksandr Yefanov[1], Lucrezia Catapano[4,5], Alessandra Henkel[1], Max O. Wiedorn[1], Olga Lorbeer[6], Eva Crosas[6], Jan Meyer[6], Valerio Mariani[1,$$$], Martin Domaracky[1], Thomas A. White[6], Holger Fleckenstein[1], Iosifina Sarrou[1,$$$$], Nadine Werner[3], Christian Betzel[3,7], Holger Rohde[2], Martin Aepfelbacher[2], Henry N. Chapman[1,7,8], Markus Perbandt[3], Roberto A Steiner[4,9#], Dominik Oberthür[1,#]

1 Center for Free-Electron Laser Science CFEL, Deutsches Elektronen-Synchrotron DESY, Notkestr. 85, 22607 Hamburg, Germany

2 Institute for Medical Microbiology, Virology and Hygiene, University Medical Center Hamburg-Eppendorf, Martinistrasse 52, 20246, Hamburg, Germany

3 Institute of Biochemistry and Molecular Biology, Laboratory for Structural Biology of Infection and Inflammation, University of Hamburg, c/o DESY, Build. 22a. Notkestr. 85, 22603 Hamburg, Germany

4 Randall Centre of Cell and Molecular Biophysics, King's College London, UK.

5 MRC Laboratory of Molecular Biology, Francis Crick Avenue, Cambridge CB2 0QH, United Kingdom

6 Deutsches Elektronen-Synchrotron DESY, Notkestr. 85, 22607 Hamburg, Germany

7 Hamburg Centre for Ultrafast Imaging, Universität Hamburg, Luruper Chaussee 149, 22761 Hamburg, Germany.

8 Department of Physics, University of Hamburg, Luruper Chaussee 149, 22761 Hamburg, Germany.

9 Dept of Biomedical Sciences, University of Padova, via Ugo Bassi 58/B, 35131 Padova, Italy.

Current Affiliation:

$ School of Applied and Engineering Physics, Cornell University, Ithaca, New York 14853, USA

$$ Department of Biochemistry, Bahauddin Zakariya University, Multan-60800, Punjab, Pakistan

$$$ Linac Coherent Light Source, SLAC National Accelerator Laboratory, Menlo Park, CA, 94025, USA

$$$$ Wiley-VCH, Berlin

* KAZ, AP and HA contributed equally to this work

# to whom correspondence should be addressed: dominik.oberthuer@desy.de, roberto.steiner@kcl.ac.uk or roberto.steiner@unipd.it

Supplementary Information

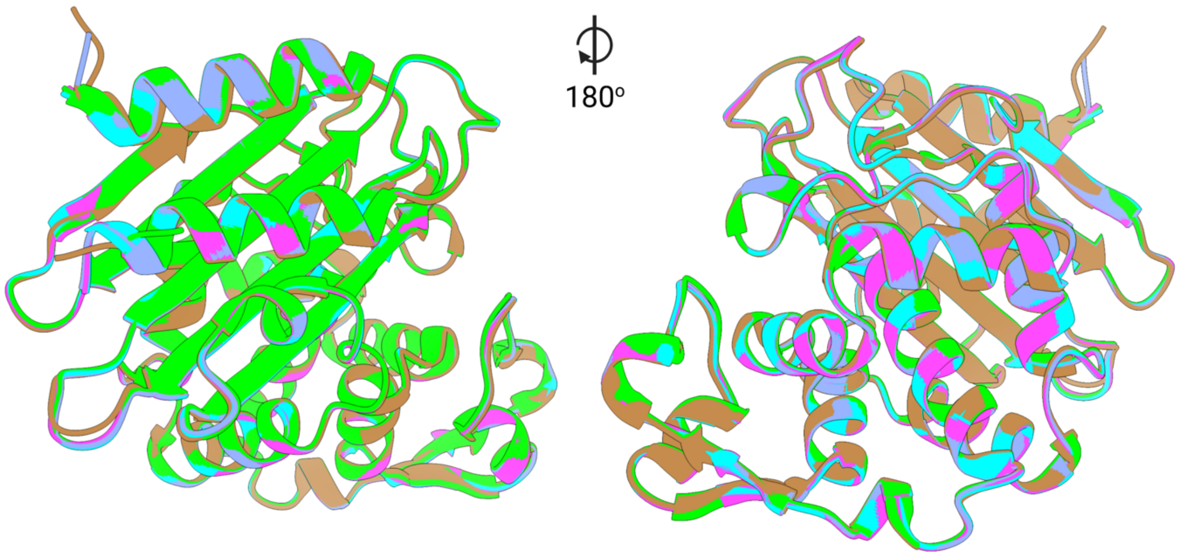

Figure S1 Aligned CTX-M-14 β-lactamase structures, cyan: 5K dataset; purple: 10K dataset; magenta: full dataset, brown: the cryoMX structure (PDB accession code: 7q0z) and green: the structure from EuXFEL (PDB accession code: 6GTH)

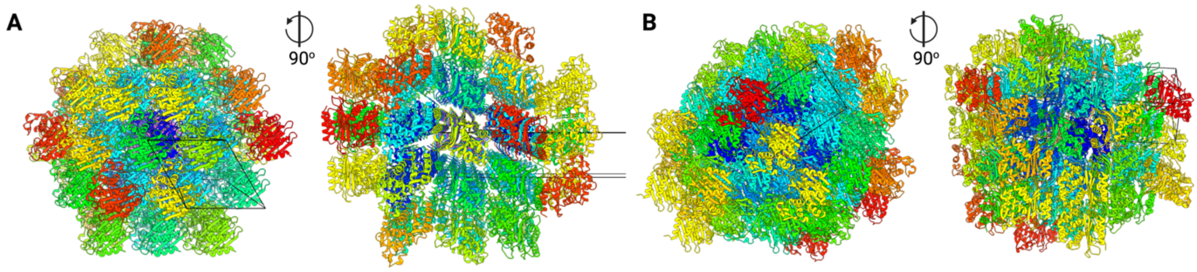

Figure S2 Crystal packing of CTX-M-14 β-lactamase. A) from RT-SSX (Spacegroup P 32 2 1) and B) from cryoMX (PDB accession code: 7q0z, spacegroup P 21 21 21), unit cell outlined in black.

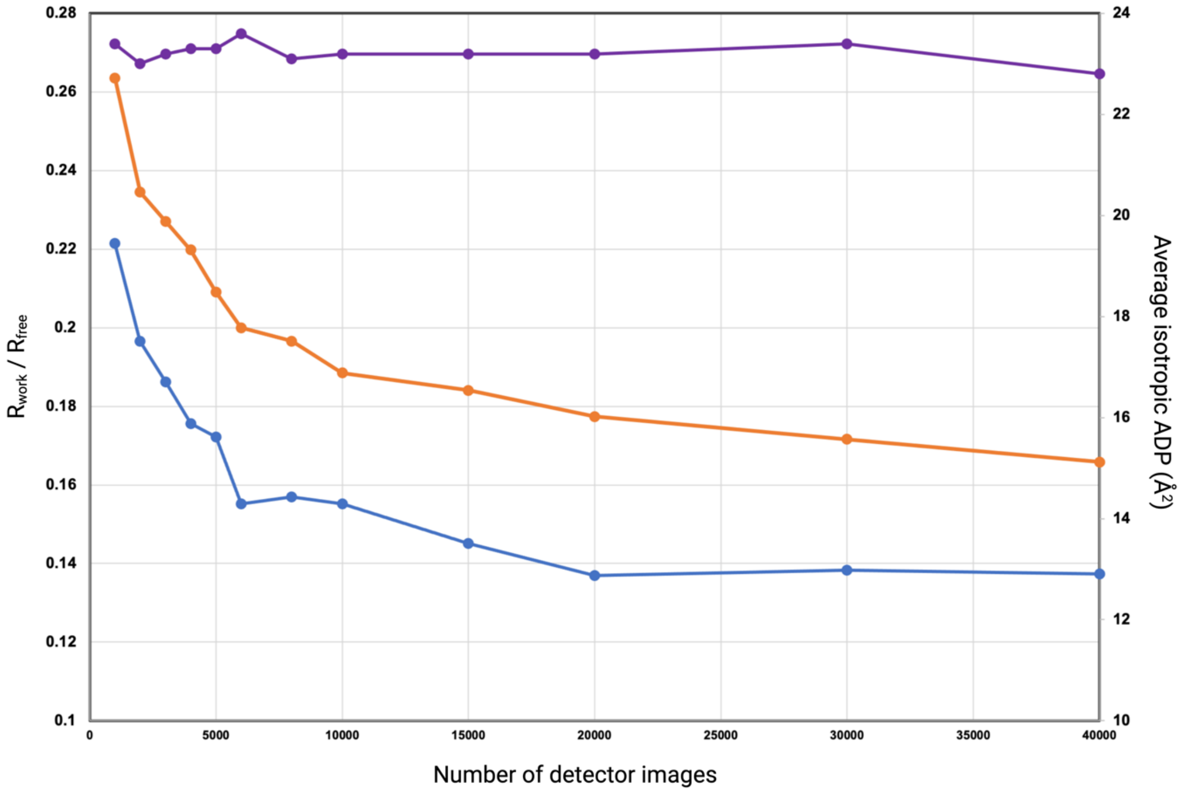

Figure S3 Plot of the evolution of Rfree (orange) Rwork (blue) and the average isotropic ADP (purple) with addition of detector images in automatic refinement of NhGH11 xylanase.

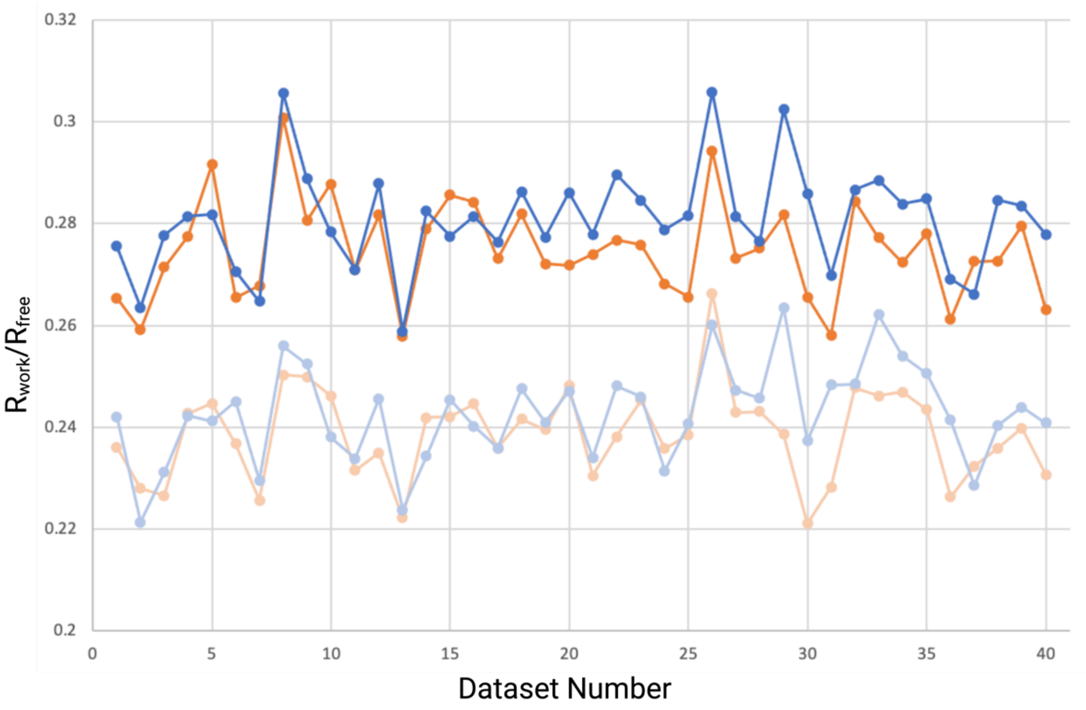

Figure S4 R_{work}/R_{free}-values for all 40 datasets, each consisting of 1000 recorded detector-frames within one run. Results from processing without partiality modelling are depicted in blue (R_{free}) and light blue (R_{work}), values from processing with partiality modelling are shown in orange (R_{free}) and ligh orange (R_{work}).

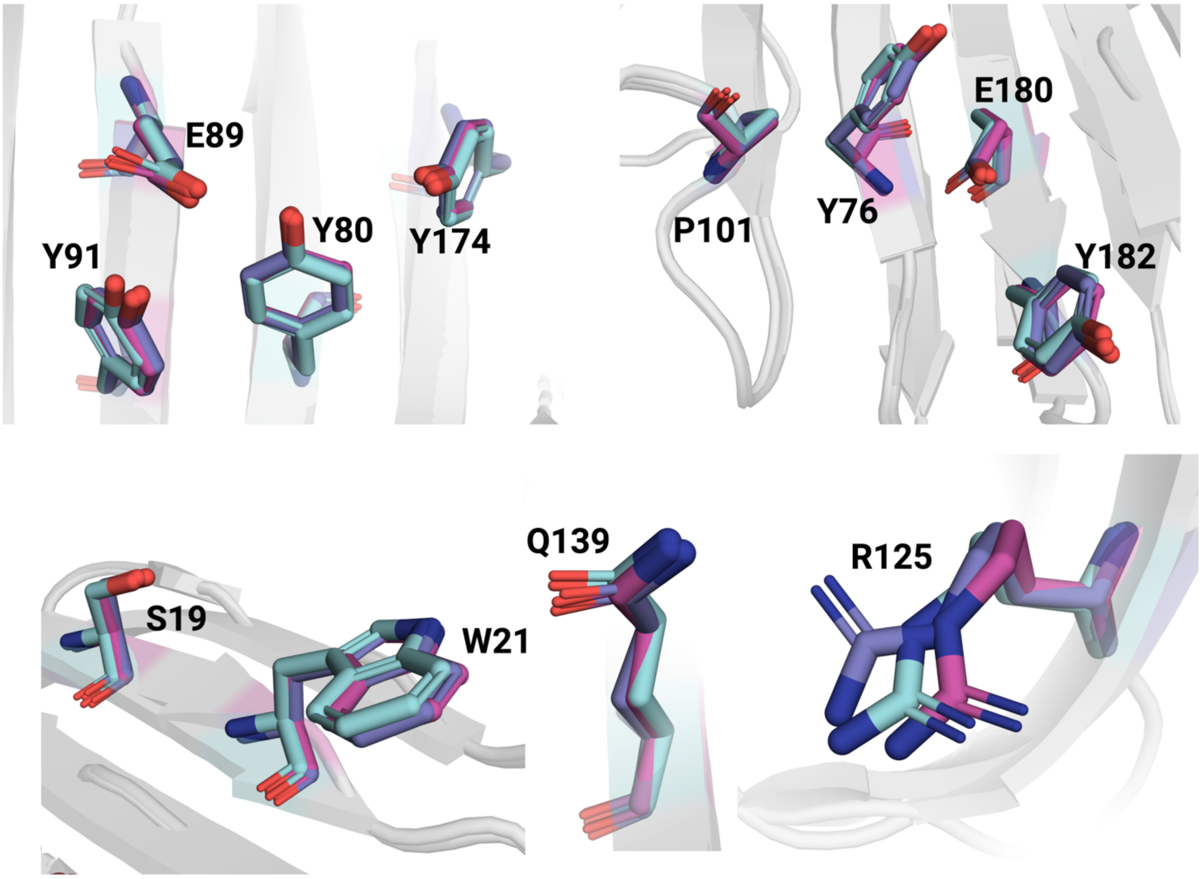

Figure S5 Detailed comparison of the spatial positions of the active site residues of NhGH11 xylanase. The full (40'000 detector images) dataset residues are shown in magenta, the 1000 detector images dataset residues in purple and the cryoMX residues (PDB accession code: 6y0h) are shown in cyan.

(*a*)

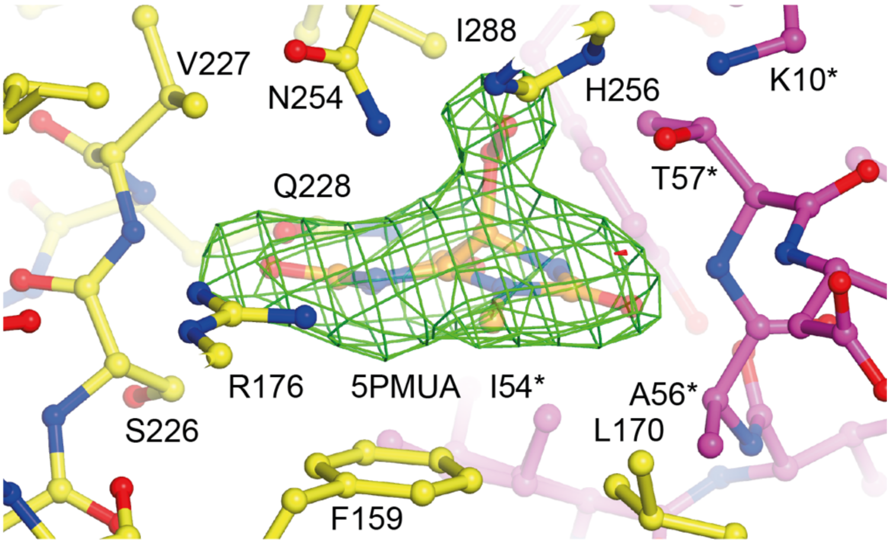

(*b*)

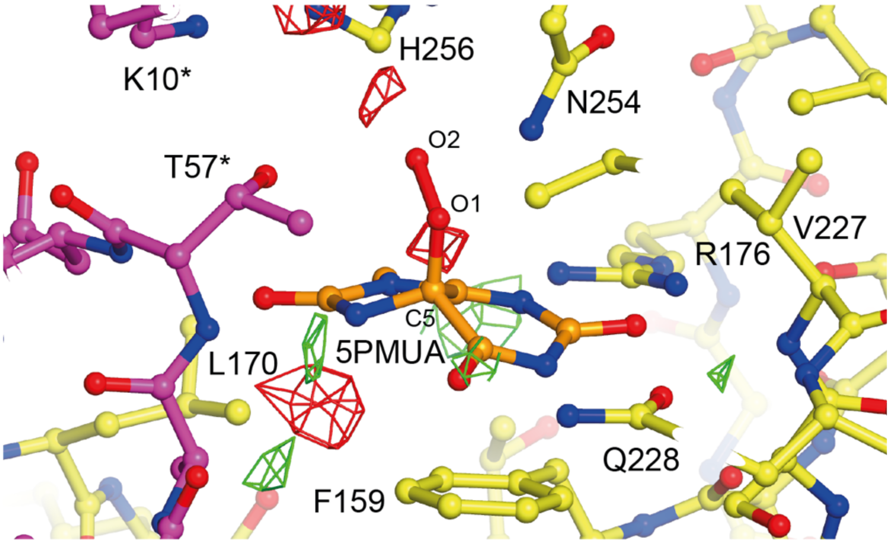

Figure S6 (a) mF_o-DF_c electron density contoured at the +3σ level (in green) for 5PMUA prior to its incorporation in refinement. The transparent ball-and-stick 5PMUA structure is shown only for reference. (b) mF_o-DF_c electron density contoured at the +2.5σ (green) and -2.5σ (red) level in the vicinity of 5PMUA (4 Å distance) after its incorporation in refinement. Although the is some negative difference density close to the 5PMUA C5-O1 bond this appears to be at the level of general map noise. Active site views in (a) and (b) are rotated by approximately 180 degrees. The color scheme is the same as that of Figure 9 of the main text.

Raddose-3D script for the X-ray dose calculation of the UOX-5PMUA complex

```
################################################################################
#                                Crystal Block                                 #
################################################################################
Crystal
Type Cuboid
PixelsPerMicron 2
Dimensions 15 15 15
AbsCoefCalc RD3D
UNITCELL 79.8 96.0 105.2 90 90 90
ANGLEP 0
ANGLEL 0
NumMonomers 8
NumResidues 301
ProteinHeavyAtoms S 8
SolventFraction 0.59
################################################################################
#                                Beam Block                                    #
################################################################################
Beam
Type Gaussian
FWHM 9 4
Collimation Rectangular 27 12
FLUX 7.9e12
ENERGY 12.0
################################################################################
#                                Wedge Block                                   #
################################################################################
Wedge 0 1
ExposureTime 3.6E-3
ANGULARRESOLUTION 0.01
STARTOFFSET -1.8 0 0
TRANSLATEPERDEGREE 3.6 0 0
ROTAXBEAMOFFSET 0
```

Results of automated refinement of NhG11 xylanase datasets:

Table S1: statistics for datasets from 1000 to 40000 detector images merged

	indexed	indexing rate	SNR	SNRhigh	Rsplit	Rsplithigh	Ccstar	Ccstar high	Time	Time Min:S	Indexed/s	Time per dataset (s)w/ Eiger (x5)	datasets/hour pilatus	datasets/ hour eiger	res at which ccstar < 50
1000	1179	117.90	1.71	0.52	55.24	226.04	0.926	0.467	40	00:00:40	29.5	8	90	450	1.83
2000	2243	112.15	2.26	0.67	41.84	165.66	0.955	0.557	80	00:01:20	28.0	16	45	225	1.73
3000	3303	110.10	2.66	0.74	35.7	148.48	0.965	0.596	120	00:02:00	27.5	24	30	150	1.73
4000	4402	110.05	3.03	0.85	30.02	126.1	0.976	0.648	160	00:02:40	27.5	32	22.5	112.5	1.65
5000	5397	107.94	3.37	0.94	27.66	113.59	0.979	0.718	200	00:03:20	27.0	40	18	90	1.65
6000	6595	109.92	3.70	1.06	25.22	100.42	0.983	0.754	240	00:04:00	27.5	48	15	75	1.65
8000	8732	109.15	4.21	1.23	22.52	85.95	0.987	0.805	320	00:05:20	27.3	64	11.25	56.25	1.65
10000	10807	108.07	4.66	1.37	20.42	78.74	0.989	0.827	400	00:06:40	27.0	80	9	45	1.58
15000	16108	107.39	5.65	1.67	16.37	62.54	0.993	0.878	600	00:10:00	26.8	120	6	30	1.58
20000	20767	103.84	6.44	1.91	14.29	54.07	0.995	0.905	800	00:13:20	26.0	160	4.5	22.5	1.53
30000	31304	104.35	7.77	2.32	13.78	44.66	0.993	0.931	1200	00:20:00	26.1	240	3	15	1.53
40000	41508	103.77	8.96	2.64	10.8	41.94	0.997	0.934	1600	00:26:40	25.9	320	2.25	11.25	1.5

Table S2: refinement statistics (automated refinement with 1.9 Å resolution cut-off)

	R	Rfree	rmsd bond	rmsd angle	b avg	N water
1000	0.2214	0.2635	0.002	0.56	23.4	285
2000	0.1966	0.2345	0.002	0.55	23	279
3000	0.1862	0.2271	0.002	0.55	23.2	264
4000	0.1756	0.2198	0.002	0.56	23.3	269
5000	0.1722	0.2091	0.002	0.56	23.3	279
6000	0.1552	0.2	0.005	0.7	23.6	271
8000	0.157	0.1966	0.003	0.63	23.1	255
10000	0.1552	0.1885	0.003	0.64	23.2	244
15000	0.1451	0.1841	0.004	0.66	23.2	235
20000	0.137	0.1774	0.006	0.79	23.2	222
30000	0.1383	0.1716	0.003	0.66	23.4	211
40000	0.1374	0.1658	0.003	0.63	22.8	217

Table S3: data quality indicators from CrystFEL for all 40 individual datasets, with (*part) and without partiality refinement.

STREAM	N ind	SNR	SNRpart	CC	Ccpart	Ccstar	Ccstarpart	devccstarpart-	deltaCCstar	Rsplit	Rsplitpart	ind rate
0	1161	1.68	2.24	0.728419	0.788536	0.918081	0.939025	3.84189E-08	-0.0209436	54.53%	45.22%	116.1
1	1179	1.71	2.27	0.753114	0.809658	0.926916	0.945949	5.06959E-05	-0.0190327	55.24%	47.06%	117.9
2	1064	1.7	2.52	0.731231	0.810832	0.919104	0.946327	5.62291E-05	-0.0272232	55.30%	44.62%	106.4
3	1060	1.53	2.62	0.701929	0.787507	0.90822	0.938682	2.1548E-08	-0.0304618	59.75%	47.99%	106
4	1099	1.62	2.57	0.701553	0.783741	0.908077	0.937423	1.9771E-06	-0.0293455	56.08%	47.60%	109.9
5	1079	1.72	2.28	0.729726	0.809312	0.918557	0.945837	4.9115E-05	-0.0272798	57.62%	46.01%	107.9
6	1114	1.67	2.69	0.719489	0.817293	0.914803	0.9484	9.1608E-05	-0.0335968	56.52%	46.78%	111.4
7	1067	1.65	2.26	0.695019	0.788694	0.905578	0.939077	6.17063E-08	-0.033499	58.42%	47.83%	106.7
8	1070	1.58	2.17	0.678492	0.77516	0.899141	0.934527	1.85028E-05	-0.0353861	59.65%	48.11%	107
9	1028	1.59	2.03	0.674061	0.731401	0.897385	0.919166	0.000386614	-0.0217811	58.39%	48.54%	102.8
10	1113	1.77	2.26	0.72317	0.816031	0.91616	0.947996	8.40487E-05	-0.0318364	55.30%	44.17%	111.3
11	1044	1.54	2.18	0.724172	0.775152	0.916528	0.934525	1.85252E-05	-0.0179965	56.45%	47.03%	104.4
12	1215	1.82	2.42	0.704513	0.826969	0.9092	0.951468	0.000159742	-0.0422675	53.29%	44.63%	121.5
13	1022	1.68	2.25	0.700711	0.801293	0.907756	0.943232	1.93891E-05	-0.0354759	56.19%	45.25%	102.2
14	966	1.65	2.16	0.661989	0.753844	0.892537	0.927172	0.000135881	-0.0346348	58.24%	48.58%	96.6
15	988	1.6	2.04	0.70043	0.780435	0.907649	0.936311	6.33676E-06	-0.0286623	59.17%	47.49%	98.8
16	1015	1.59	2.15	0.744897	0.816045	0.924013	0.948001	8.41276E-05	-0.0239877	57.34%	46.53%	101.5
17	946	1.62	1.95	0.715974	0.777626	0.913499	0.935363	1.20117E-05	-0.0218638	59.20%	48.50%	94.6
18	937	1.52	2.27	0.698589	0.774572	0.906947	0.934327	2.02625E-05	-0.0273802	57.90%	46.52%	93.7
19	978	1.63	2.51	0.673828	0.757838	0.897293	0.928568	0.00010528	-0.031275	60.74%	49.75%	97.8
20	1108	1.68	2.4	0.727726	0.798273	0.917828	0.942243	1.16582E-05	-0.024415	56.19%	46.92%	110.8
21	917	1.69	2.62	0.708745	0.767531	0.910796	0.931922	4.77024E-05	-0.0211259	58.04%	47.23%	91.7
22	1075	1.78	2.2	0.717139	0.806467	0.913932	0.944916	3.70577E-05	-0.0309841	55.95%	46.50%	107.5
23	1056	1.71	2.4	0.737302	0.793715	0.921298	0.940742	3.66189E-06	-0.0194442	56.03%	46.04%	105.6
24	1032	1.57	2.08	0.734921	0.794798	0.92044	0.9411	5.15702E-06	-0.0206595	58.47%	47.93%	103.2
25	957	1.5	2.2	0.65056	0.783659	0.887857	0.937395	2.05461E-06	-0.0495382	60.77%	49.58%	95.7
26	1013	1.63	2.44	0.699672	0.786927	0.90736	0.938488	1.15799E-07	-0.0311283	58.24%	47.68%	101.3
27	1120	1.67	2.26	0.71926	0.785178	0.914718	0.937904	8.55611E-07	-0.0231856	55.45%	46.75%	112
28	1011	1.63	1.95	0.686392	0.767871	0.902239	0.932039	4.6104E-05	-0.0297996	60.29%	48.36%	101.1
29	1064	1.74	3.4	0.706976	0.809865	0.91013	0.946015	5.16502E-05	-0.0358854	54.49%	45.37%	106.4
30	967	1.54	2.38	0.732241	0.826149	0.919471	0.951209	0.000153279	-0.0317382	57.40%	45.03%	96.7
31	1025	1.68	2.3	0.743212	0.823773	0.923414	0.950459	0.000135266	-0.027045	57.00%	46.50%	102.5
32	915	1.54	2.19	0.665545	0.721936	0.893975	0.915706	0.000534673	-0.0217306	60.24%	49.20%	91.5
33	918	1.45	2.06	0.681334	0.76818	0.90026	0.932145	4.46758E-05	-0.0318846	61.00%	50.17%	91.8
34	983	1.62	1.83	0.688741	0.787371	0.903153	0.938636	3.70149E-08	-0.0354832	59.37%	46.83%	98.3
35	1013	1.64	2.27	0.719488	0.796984	0.914802	0.94182	8.94613E-06	-0.0270176	57.22%	47.14%	101.3
36	1037	1.67	2.33	0.68656	0.793084	0.902305	0.940534	2.90671E-06	-0.0382285	57.09%	47.39%	103.7
37	1006	1.62	2.88	0.686336	0.78667	0.902218	0.938403	1.8147E-07	-0.0361846	57.16%	46.14%	100.6
38	1132	1.65	2.07	0.720341	0.782083	0.915117	0.936866	3.85177E-06	-0.021749	57.17%	49.13%	113.2
39	1014	1.62	2.02	0.705135	0.771351	0.909435	0.93323	3.13454E-05	-0.0237949	58.78%	50.39%	101.4

Table S4 refinement statistics (automated refinement) of all 40 datasets (1000 detector images each).

Partiality Refined							Non-partiality refined							
R	Rfree	rmsd bond	rmsd angle	b avg	N water		R	Rfree	rmsd bon	rmsd angle	b avg	N water	deltaRfree	delta r
0.2361	0.2654	0.002	0.56	22	283		0.2421	0.2756	0.002	0.54	23.6	286	-0.0102	-0.006
0.228	0.2591	0.002	0.56	22	281		0.2214	0.2635	0.002	0.56	23.4	285	-0.0044	0.0066
0.2266	0.2715	0.002	0.56	19.7	299		0.2312	0.2777	0.002	0.54	20.8	298	-0.0062	-0.0046
0.2427	0.2775	0.002	0.54	20.4	254		0.2423	0.2814	0.002	0.56	20.5	291	-0.0039	0.0004
0.2447	0.2916	0.002	0.55	18.9	283		0.2412	0.2818	0.002	0.54	21.9	299	0.0098	0.0035
0.2368	0.2655	0.003	0.59	20.2	285		0.2451	0.2706	0.003	0.59	21.4	282	-0.0051	-0.0083
0.2257	0.2678	0.002	0.57	19.1	294		0.2296	0.2647	0.002	0.55	20.8	279	0.0031	-0.0039
0.2503	0.3008	0.002	0.53	18.9	264		0.256	0.3057	0.002	0.54	20.2	215	-0.0049	-0.0057
0.2499	0.2806	0.002	0.54	19.4	239		0.2524	0.2888	0.002	0.54	21.4	275	-0.0082	-0.0025
0.2462	0.2878	0.002	0.59	20.5	260		0.2381	0.2784	0.002	0.55	21.9	271	0.0094	0.0081
0.2315	0.2709	0.002	0.52	21.2	237		0.2339	0.271	0.002	0.54	22.1	224	-0.0001	-0.0024
0.235	0.2817	0.002	0.55	19.7	266		0.2456	0.2879	0.002	0.56	21.2	273	-0.0062	-0.0106
0.2222	0.2579	0.002	0.53	21.1	245		0.2238	0.2589	0.002	0.53	22.4	241	-0.001	-0.0016
0.2419	0.2789	0.002	0.55	20.5	252		0.2343	0.2825	0.002	0.53	21.9	226	-0.0036	0.0076
0.2421	0.2856	0.002	0.53	19.4	255		0.2454	0.2775	0.002	0.55	21.7	245	0.0081	-0.0033
0.2446	0.2842	0.002	0.55	19.1	293		0.2402	0.2814	0.002	0.56	21.4	252	0.0028	0.0044
0.2359	0.2731	0.002	0.53	24.3	225		0.2359	0.2764	0.002	0.53	24.3	254	-0.0033	0
0.2416	0.2819	0.003	0.61	19.3	278		0.2476	0.2863	0.002	0.55	20.9	274	-0.0044	-0.006
0.2396	0.2721	0.002	0.57	20.3	301		0.241	0.2773	0.002	0.58	22.2	288	-0.0052	-0.0014
0.2481	0.2718	0.002	0.52	20.7	259		0.2471	0.2861	0.002	0.53	20.4	283	-0.0143	0.001
0.2305	0.274	0.002	0.55	20.4	287		0.2341	0.2779	0.003	0.58	21.6	283	-0.0039	-0.0036
0.2382	0.2768	0.002	0.54	18.7	260		0.2481	0.2896	0.002	0.58	20.4	288	-0.0128	-0.0099
0.2453	0.2758	0.002	0.55	21.4	202		0.246	0.2846	0.002	0.52	21.4	227	-0.0088	-0.0007
0.2358	0.2682	0.002	0.55	18.8	284		0.2314	0.2788	0.002	0.55	20.8	263	-0.0106	0.0044
0.2385	0.2656	0.002	0.57	20.3	299		0.2407	0.2816	0.002	0.54	21.8	221	-0.016	-0.0022
0.2663	0.2943	0.002	0.54	19.7	221		0.2601	0.3059	0.002	0.54	20.2	259	-0.0116	0.0062
0.2429	0.2732	0.002	0.55	19	239		0.2473	0.2814	0.002	0.56	20.8	288	-0.0082	-0.0044
0.2431	0.2752	0.002	0.54	19	254		0.2457	0.2765	0.002	0.56	22.7	237	-0.0013	-0.0026
0.2386	0.2817	0.002	0.53	21	225		0.2635	0.3024	0.002	0.53	21.1	221	-0.0207	-0.0249
0.2211	0.2656	0.002	0.54	21.8	289		0.2373	0.2859	0.002	0.56	20.8	278	-0.0203	-0.0162
0.2283	0.2581	0.002	0.56	21	309		0.2483	0.2699	0.002	0.57	21.6	292	-0.0118	-0.02
0.2478	0.2844	0.002	0.55	20.2	237		0.2485	0.2867	0.002	0.54	21.2	240	-0.0023	-0.0007
0.2462	0.2772	0.002	0.56	22.3	235		0.2621	0.2885	0.002	0.59	21.4	228	-0.0113	-0.0159
0.2469	0.2724	0.002	0.56	20	302		0.254	0.2839	0.002	0.55	23.6	268	-0.0115	-0.0071
0.2435	0.2781	0.002	0.57	18.6	253		0.2506	0.2849	0.002	0.57	20.2	291	-0.0068	-0.0071
0.2263	0.2612	0.002	0.57	19.8	302		0.2414	0.2691	0.002	0.56	21.7	285	-0.0079	-0.0151
0.2324	0.2726	0.002	0.55	20.3	263		0.2286	0.2661	0.002	0.55	21.2	255	0.0065	0.0038
0.2359	0.2727	0.004	0.67	20.2	219		0.2404	0.2846	0.002	0.54	21.2	263	-0.0119	-0.0045
0.2398	0.2796	0.002	0.53	21.2	234		0.2439	0.2835	0.002	0.53	21.7	231	-0.0039	-0.0041
0.2307	0.2632	0.002	0.57	19.2	283		0.2409	0.2779	0.002	0.56	20.5	233	-0.0147	-0.0102
